# Angelica Dahurica Regulated the Polarization of Macrophages and Accelerated Wound Healing in Diabetes: A Network Pharmacology Study and *In Vivo* Experimental Validation

**DOI:** 10.3389/fphar.2021.678713

**Published:** 2021-06-21

**Authors:** Yonghui Hu, Sisi Lei, Zhiyue Yan, Zhibo Hu, Jun Guo, Hang Guo, Bei Sun, Congqing Pan

**Affiliations:** ^1^ NHC Key Laboratory of Hormones and Development, Tianjin Key Laboratory of Metabolic Diseases, Chu Hsien-I Memorial Hospital and Tianjin Institute of Endocrinology, Tianjin Medical University, Tianjin, China; ^2^ The Second Clinical Medical College of Guangzhou University of Chinese Medicine, Guangzhou, China; ^3^ Department of Emergency, the Second Affiliated Hospital of Guangzhou University of Chinese Medicine, Guangzhou, China

**Keywords:** diabetes, wound healing, angelica dahurica, systems pharmacology, inflammation

## Abstract

Diabetic wounds exhibit retarded and partial healing processes. Therefore, patients are exposed to an elevated risk of infection. It has been verified that Angelica dahurica (Hoffm.) Benth. and Hook. f. ex Franch. and Sav (*A. dahurica*) is conducive for wound healing. However, the pharmacological mechanisms of *A. dahurica* are yet to be established. The present study uses network pharmacology and *in vivo* experimental validation to investigate the underlying process that makes *A. dahurica* conducive for faster wound healing in diabetes patients. 54 potential targets in *A. dahurica* that act on wound healing were identified through network pharmacology assays, such as signal transducer and activator of transcription 3 (STAT3), JUN, interleukin-1β (IL-1β), tumor necrosis factor (TNF), and prostaglandin G/H synthase 2 (PTGS2). Furthermore, *in vivo* validation showed that *A. dahurica* accelerated wound healing through anti-inflammatory effects. More specifically, it regulates the polarization of M1 and M2 subtypes of macrophages. *A. dahurica* exerted a curative effect on diabetic wound healing by regulating the inflammation. Hence, pharmacologic network analysis combined with *in vivo* validation elucidated the probable effects and underlying mechanisms of *A. dahurica*’s therapeutic effect on diabetic wound healing.

## Introduction

A wound heals in a natural reparative process. It is the process through which the body responds to tissue injuries. Wound healing comprises four main continuous and overlapping stages, namely homeostasis, inflammation, proliferation, and tissue remodeling (Gurtner et al., 2008). However, failing to advance these four stages in an orderly manner results in impaired wound healing, representing one of the primary causes of fatalities associated with diabetes. It affects nearly 25% of diabetes patients (Armstrong et al., 2017). Such wounds often become points of entry for bacterial infections that could result in sepsis and lower-extremity amputation (Morbach et al., 2012). Staggeringly, lower-extremity amputation cases exhibit a five-year mortality rate of nearly 50% (Gregg et al., 2014). Considering the increasing number of diabetes patients, the prevalence of wound complications is most likely to rise substantially, posing a massive financial burden on patients and the worldwide healthcare system.

The systematic complications associated with diabetes potentiate the challenges involved in designing a suitable treatment strategy (e.g., tissue hypoxia, impaired inflammatory response, and reduced collagen generation) (Wong et al., 2015). Generally, the protocol for treating a chronic wound entails debridement of necrotic tissue, controlling the infection through the use of topical antibiotics, and applying a dressing on the wound. (e.g., films, fibers, and hydrogels) (Powers et al., 2016). Health care professionals combine these medications, often with less than satisfactory outcomes. Thus, it is clinically significant to develop efficient therapeutic strategies. The best method is to identify new effective medication, which would promote wound closure and improve the prognosis of patients.

Angelica dahurica (Hoffm.) Benth. and Hook. f. ex Franch. and Sav (Bai-zhi in Chinese, *A. dahurica*) is a popular plant. It is edible and has medicinal qualities. It is in abundance in northern China. Besides its consumption as a dietary supplement, *A. dahurica* is also effective for treating pain, abscesses, and furunculosis (Hou et al., 2017). The multiple benefits of *A. dahurica* have been verified, including anti-inflammatory, proangiogenic, and cell-stimulatory activities (Guo et al., 2020) (Pereira and Bártolo, 2016). A previous study revealed that *A. dahurica* blocks excessive proinflammatory cytokines, such as interleukin-1β (IL-1β), interleukin-6 (IL-6), and interferon-γ (IFN-γ) to provide protective effects against periodontal diseases to alleviate inflammatory responses (Lee et al., 2017). Our group indicated the promoted benefits of *A. dahurica* with regards to accelerating wound healing via its effects on neovascularization in excisional wounds in diabetic mice (Zhang et al., 2017). However, the curative effects of *A. dahurica* that regulate inflammation in diabetic wound healing remain unclear. Meanwhile, there is evidence that macrophage polarization plays a crucial part in the inflammatory state and angiogenesis of wound surface (Wynn and Vannella, 2016). Therefore, we speculated that *A. dahurica* could promote diabetic wound healing by regulating macrophage polarization. Besides, *A. dahurica* has an intricate composition with over 70 biologically active metabolites, including imperatorin, isoimperatorin, oxypeucedanin hydrate, and more. (Zhao et al., 2016). Given that studies have not reported the underlying pharmacological processes of *A. dahurica* and its active ingredients clearly, it is crucial to discover innovative methods to recognize the potential treatment goals and active compounds in *A. dahurica*. Network pharmacology analysis is an advanced method. It combines drug half-life (HL) and drug-likeness (DL) calculations, multi-target prediction models, network analyses, and bioinformatics to identify active ingredients and therapeutic goals of traditional Chinese medicine (TCM) (Liu et al., 2013). Since network pharmacology aims to explore drug interference or effect on diseases and demonstrating the molecular level synergism law of multicomponent drugs, it aligns with the idea of TCM and may have potential use in investigating multi-target, complete interventions using TCM as a basis. This study involves an integrative study based on the examination of the complete formula up to the active constituents to facilitate an unequivocal interpretation of the therapeutic effects of *A. dahurica* in diabetic wound healing. The study combines the network pharmacology method and *in vivo* validation.

## Methods

### Screening of Active Compounds

Each active compound in *A. dahurica* was obtained from the Traditional Chinese Medicine Systems Pharmacology database and Analysis Platform (TCMSP, http://tcmspw.com) and literature review. TCMSP refers to a specialized systems pharmacology platform dedicated to Chinese traditional herbal medications. It contains the associations between medication, targets, and illnesses (Ru et al., 2014). Oral bioavailability (OB) is a fractional dosage that reaches the circulatory system and is significant in the discovery of most oral TCM. Drug-likeness (DL) refers to a qualitative examination technique for drug design and is widely utilized to assess the suitability of a compound to be used as medication (Wu et al., 2020). Since a majority of the compounds in TCM lack sufficient pharmacological features, the compounds cannot bind explicit cell protein markers effectively. Numerous scholars have suggested that molecules with OB ≥ 30% and DL ≥ 0.18 could be regarded to possess improved pharmacological effects and are suitable candidate compounds for extensive examinations (Yu et al., 2018; Kim et al., 2019; Ma et al., 2019; Wang et al., 2019). Thus, the present empirical examination utilized this technique for screening compounds and identifying ones suitable for additional analysis.

### Identification of Wound Healing Targets

The authors searched the following databases to identify the therapeutic targets for treating wound healing: a) Gene Cards database (https://www. genecards. org/, version 4.9.0) (Safran et al., 2010); b) Online Mendelian Inheritance in Man (OMIM, http://omim.org/) database (Hamosh et al., 2005). The present research adopted the “Wound healing” as the keyword to search the databases. The corresponding genes for diseases linked with wound healing were obtained accordingly.

### Compounds-Genes Network Construction

Firstly, the attained drug targets were intersected with the disease genes to obtain the intersected genes. Afterward, according to the interactions between the compounds and target genes, a network of complex information was established. Next, the Cytoscape 3.7.2 software (https://cytos cape. Org/index. html) (Shannon et al., 2003) was employed to conduct a visual analysis of the “Compounds-Genes Network.” The Cytoscape 3.7.2 software is a dedicated graphing display, network analysis, and editing software.

### PPI Network Construction

Data corresponding to the protein-protein interactions was collected from the String database (https://string-db.org/, version 11.0, updated on January 19, 2019) (Hsia et al., 2015). Once all the related ingredients and their probable targets were obtained, the data was exported onto the String database to locate the likely intersecting points and establish the association between the targets. Afterward, the output files were used as input to the Cytoscape software to obtain protein-protein interaction (PPI) complex diagrams in a representable form to characterize the association between *A. dahurica* and wound healing intersection genes.

### Network Analysis

Gene ontology (GO) enrichment analysis and Kyoto Encyclopedia of Genes and Genomes (KEGG) pathway enrichment were carried out by connecting targets to the Metascape database (https://metascape.org/gp/index.html#/main/step1). In the GO evaluation, the GO term “biological process” was highlighted. P- values were derived from the Metascape database and allocated to below 0.05. The smaller the *p*-value, the higher the enrichment.

### Chemical Identification and Murine Model of Wound Healing.

The herbal concentrate granules of *A. dahurica* were purchased from Yifang Pharmaceutical. INC. (Guangdong. China). Lot number: CG1802003.

Concentrated granules containing 100 mg of *A. dahurica* were mixed with 1 ml of 50% methanol. Ultrasonic extraction was conducted for a duration of 1 min. Next, the samples were centrifuged at 10,000 rpm for 10 min at a temperature of 4°C. The filtrate was diluted at 0.2–1 ml, and mixed with ultrasonic for 1 min. Next, the sample was once more centrifuged at 10,000 rpm for a duration of 10 min at a temperature of 4°C. Next, 1 μL of an aliquot of the supernatant was injected into the UPLC-QTOF/MS system.

UPLC-Q-TOF-MS analysis was separated on a Waters ACQUITY UPLC BEH C_18_ (2.1 mm × 50 mm, 1.7 μm) column, eluted with acetonitrile-water gradient elution. The flow rate was 0.25 ml/min. The volume of the injection was 1 μL. The column oven’s temperature was set to 30°C. The UPLC/Q-TOF-MS system had an electrospray ion source that operated in a positive ESI mode. The following MS parameters are related to the process: source temperature of 100°C, 250°C for desolvation temperature, cone gas flow of 50 L/h, desolvation gas of 600 L/h, 3.0 kV for capillary voltage, and collision energy of 30–50V, respectively. The chromatogram of *A. dahurica* extract is shown in Supplementary Figure S1 **.**

This study used db/db (C57BLKS/J-leprdb/leprdb) male mice aged eight weeks as a model for Type-2 Diabetes Mellitus (T2DM). The mean fasting blood glucose (FBG) of db/db mice was 27.41 ± 1.02 mmol/L. Meanwhile, the control group comprised heterozygous control db/m mice. The FBG of db/m mice was 7.45 ± 0.14 mmol/L. The present study was approved by the Experimental Animal Ethical Committee of Tianjin Medical University. Before the tests, the mice were kept in specific pathogen-free settings. The Leprdb/db mice were randomly allocated into two groups, assigned to the experimental control group (db/db, *n* = 18) and experimental group (db/db + *A. dahurica*, *n* = 18). The mice in the experimental group were treated with concentrate granules of *A. dahurica* dosage of 1.8 g/kg by gastric gavage per day. Meanwhile, Leprdb/m mice (db/m, *n* = 18) and db/db experimental control groups were administered with equal amounts of physiological saline once per day. After anesthetizing the mice with an intraperitoneal injection containing sodium pentobarbital, the dorsum was shaved and sterilized with antiseptic wipes. Two full-thickness wounds that were 6-mm in diameter were excised. Post-injury, six mice from each group were euthanized using an intraperitoneal injection containing sodium pentobarbital on day 4, and six were euthanized on day 7. The wounds were excised using scissors. One tissue from each mouse was treated with formalin and processed to use for histological analysis, and the other was snap-frozen at a temperature of −80°C.

On days 0, 2, 4, 7, and 14 after wounding, the mice were anesthetized with an intraperitoneal injection of sodium pentobarbital. The wounds were photographed to monitor the healing process. The area of the wounds was analyzed by tracing the wound margin with a fine-resolution computer mouse in an NIH ImageJ analyzer to calculate the pixel area. The following equation was used to determine the wound closure: wound closure (%) = (A0—At)/A0 × 100, where A0 denotes the area of the wound immediately after the surgery and At represents the wound area on days 0, 2, 4, 7, and 14 post-surgery.

### Histology and Immunofluorescence Staining

Skin ulcers and surrounding tissues were collected from the mice on day 4 and day 7, and treated with 10% formaldehyde and fixed in paraffin. Small sections from the wound (4-μm thick) were deparaffinized with xylene and rehydrated through a graded ethanol series and stained with H&E (hematoxylin and eosin). CD31, an indicator of the expression of endothelial cells in preliminary vascular development, was utilized to distinguish newly formed blood vessels. Immunofluorescence double-staining of CD68 and inducible nitric oxide synthase (iNOS), a marker of pro-inflammatory M1 phenotype of macrophage and immunofluorescence double-staining of CD68, and Arginase-1 (Arg-1), a marker of anti-inflammatory M2 phenotypes of macrophage, were utilized to identify the phenotypes of macrophage. Regarding immunofluorescence, the skin sections collected on day 4 were heated with Tris-EDTA, perforated with 1% Triton x-100, and blocked with 5% BSA. It was followed by overnight incubation at 4°C with primary antibodies: anti-CD68 antibody (1:50, ab955, Abcam, United States), anti-iNOS antibody (1:50, ab15323, Abcam, United States), anti-Arg-1 antibody (1:50, ab91279, Abcam, United States), anti-CD31 antibody (1:50, ab28364, Abcam, United States). Alexa Fluor 488 conjugated goat anti-mouse IgG second antibodies and Alexa Fluor 594 conjugated goat anti-rat IgG second antibodies (all 1:200, Sungene, Tianjin, China) were included on the specimens for a duration of 1 h at room temperature (RT). Besides, the specimens were stained with 4′, 6-diamidino-2-phenylindole (DAPI) to visualize the nuclei. Fluorescence signals were detected with a light microscope.

### Western Blot Analysis

A RIPA buffer (Solarbio, China) was used to extract proteins from skin tissues per the manufacturer’s instructions. Moreover, a BCA protein assay kit (Solarbio, China) was employed to obtain the protein concentrations. The proteins from each empirical group were separated by SDS-PAGE gels, shifted onto nitrocellulose (NC) membranes, blocked with 5% defatted milk for 2 h at RT, and incubated overnight at a temperature of 4°C with the following primary antibodies: anti-iNOS antibody (1:500, ab15323, Abcam, United States), anti-Arg-1 antibody (1:500, ab91279, Abcam, United States), anti- IL-1β antibody (1:500, ab9722, Abcam, United States), anti-TNF-α (1:500, ab1793, Abcam, United States), anti-VEGF (vascular endothelial growth factor) antibody (1:1000,19003-1-AP, Proteintech, China), anti-CD68 antibody (1;1000, ab955, Abcam, United States), anti- prostaglandin G/H synthase 2 (PTGS2) antibody (1:500, sc-1747, Santa Cruz Biotechnology, Germany), anti-transforming growth factor-beta1 (TGF-β1) antibody (1;1000, ab170874, Abcam, United States), anti-IL-6 antibody (1:1000, ab9324, Abcam, United States), anti-signal transducer and activator of transcription 3 (STAT3) antibody (1:1000, 4904, Cell Signaling Technology, United States), anti-JUN antibody (1:1000, 24909-1-AP, Proteintech, China) and anti-GAPDH (1:4000, 60004-1-Ig, Proteintech, China). After washing, the HRP-conjugated secondary anti-rabbit/mouse antibody (Sungene Biotech, China) was utilized at a 1:3000 dilution ratio for a 1-h period at RT. A visualization of the bands of protein was obtained using an enhanced chemiluminescence (ECL) kit (Advansta, United States). The ImageJ software was used to quantify the densitometry analyses. The housekeeping protein GAPDH was utilized as a loading control.

### Statistical Analysis

The representation of data takes place in the form of mean ± SD. A one-way analysis of variance was used as the basis to evaluate the significance of the results using Prism 8.0.1 (Graphpad, San Diego, CA, United States). *p* < 0.05 was regarded to be significant. We repeated each test for a minimum of three times.

## Results

### Screening for Active Compounds and The Related Targets of The Compounds in *A. dahurica*.

The TCMSP database lists 223 compounds of *A. dahurica*. Out of these, 20 compounds possessed an OB higher than 30.0% and a DL exceeding 0.18, which were consistent with the inclusion criteria of the study. Table 1 lists the 20 active ingredients from *A. dahurica* and their corresponding predicted OB and DL. Once the compounds were collected from the TCMSP database, they were converted into the UniProt database and the redundant items were deleted. Ultimately, we obtained 74 targets related to the compounds.

**TABLE 1:** Showing the 20 active compounds from *A. dahurica* and their corresponding predicted oral bioavailability (OB) and drug-likeliness (DL).

No	PubChem molecule ID	Molecule name	OB value (%)	DL value
BZ1	MOL001494	Mandenol	42	0.19
BZ2	MOL001939	Alloisoimperatorin	34.8	0.22
BZ3	MOL001941	Imperatorin	34.55	0.22
BZ4	MOL001942	Isoimperatorin	45.46	0.23
BZ5	MOL001956	Cnidilin	32.69	0.28
BZ6	MOL002883	Ethyl_oleate	32.4	0.19
BZ7	MOL005789	Neobyakangelico l	36.18	0.31
BZ8	MOL005792	Pabulenol	42.85	0.26
BZ9	MOL005800	Byakangelicol	41.42	0.36
BZ10	MOL005806	Oxypeucedanin hydrate	39.99	0.29
BZ11	MOL005807	Sen-byakangelicol	58	0.61
BZ12	MOL000358	Beta-sitosterol	36.91	0.75
BZ13	MOL000449	Stigmasterol	43.83	0.76
BZ14	MOL000953	Cholesterol	37.87	0.68
BZ15	MOL001749	Bis [(2R)-2-ethylhexyl] benzene-1,2-dicarboxylate (ZINC03860434)	43.59	0.35
BZ16	MOL002644	Phellopterin	40.19	0.28
BZ17	MOL003588	Alloimperatorin	36.31	0.22
BZ18	MOL003791	2-Linoleoylglycerol	37.28	0.3
BZ19	MOL007514	11,14-Eicosadienoic acid, methyl ester	39.67	0.23
BZ20	MOL013430	Prangenin	43.6	0.29

### Collecting Identified Therapeutic Gene Targets for Wound Healing

Overall, 4,129 identified therapeutic markers for wound healing were obtained from the Gene Cards database. Additionally, five more therapeutic targets were identified for wound healing were collected from the OMIM database. Once the superfluous targets were eliminated, a total of 4,134 known therapeutic markers for wound healing remained, which included STAT3, PTGS2, TNF and IL-6, etc.

### Compounds-Genes Network Analysis

**FIGURE 1:**
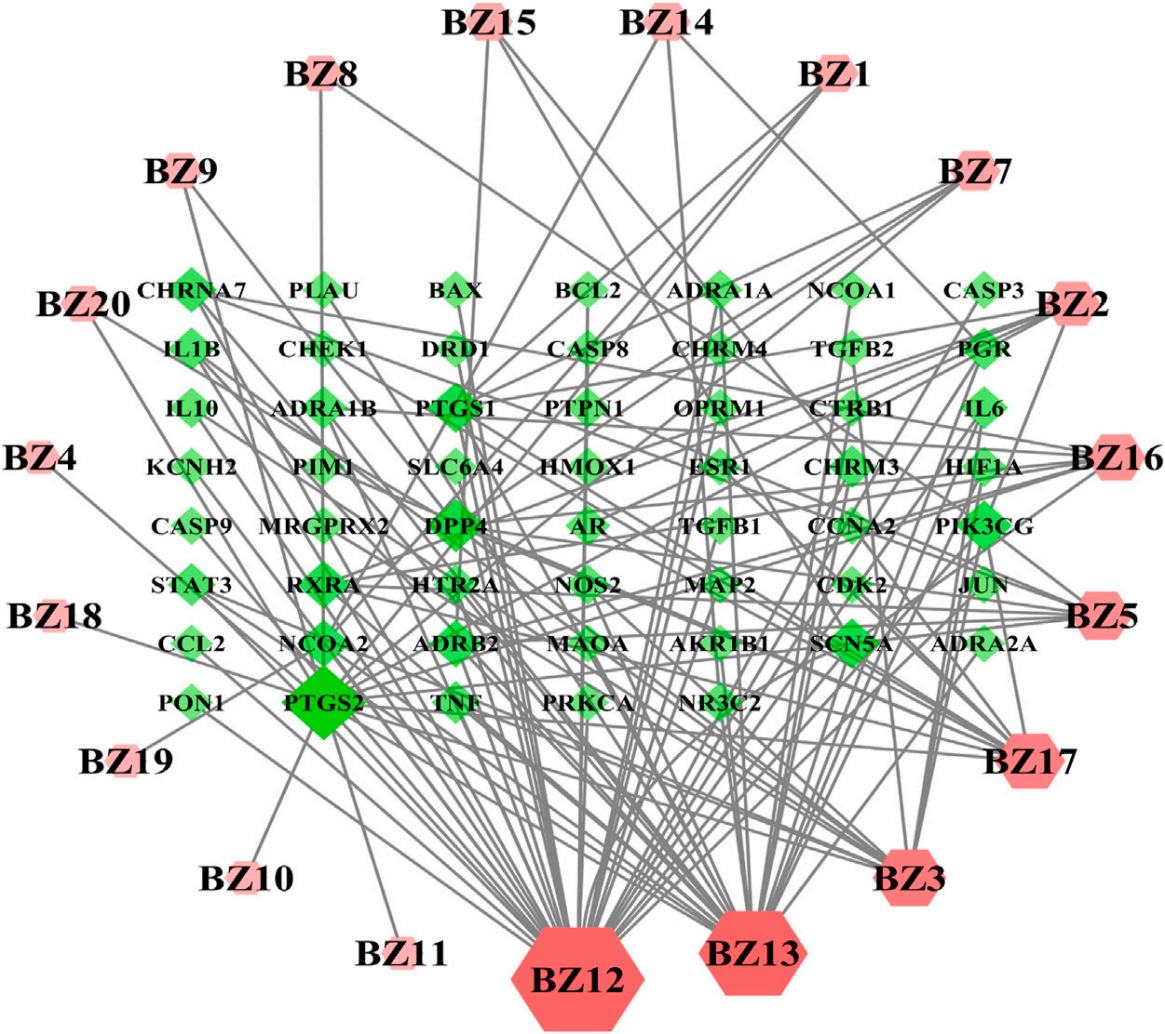
The Compounds-Genes (C–G) network.

Overall, 54 overlaps exist between the 4,134 disease genes and 74 drug genes. It means that these 54 overlapping genes could be instrumental for treating wound healing using *A. dahurica*. The “Compounds-Genes Network” shown in Figure 1 was constructed using the Cytoscape software to elucidate the likely mechanism of *A. dahurica* acting on wound healing. Since the target of the compound BZ6, also termed ethyl oleate, did not intersect with the target of wound healing, it was excluded. It was observed that out of the remaining 19 compounds,β-sitosterol (BZ12) interacts with the largest number of targets, such as IL-1β, PTGS2 and TNF. Additionally, as one of the main active ingredients of *A. dahurica* (Mi et al., 2017), it was speculated that imperatorin (BZ3) was related to PTGS2, IL-1β, IL-10, IL-6, etc. Notably, PTGS2 was the most important target node, it could be the target of numerous compounds.

### PPI Network Analysis

**FIGURE 2:**
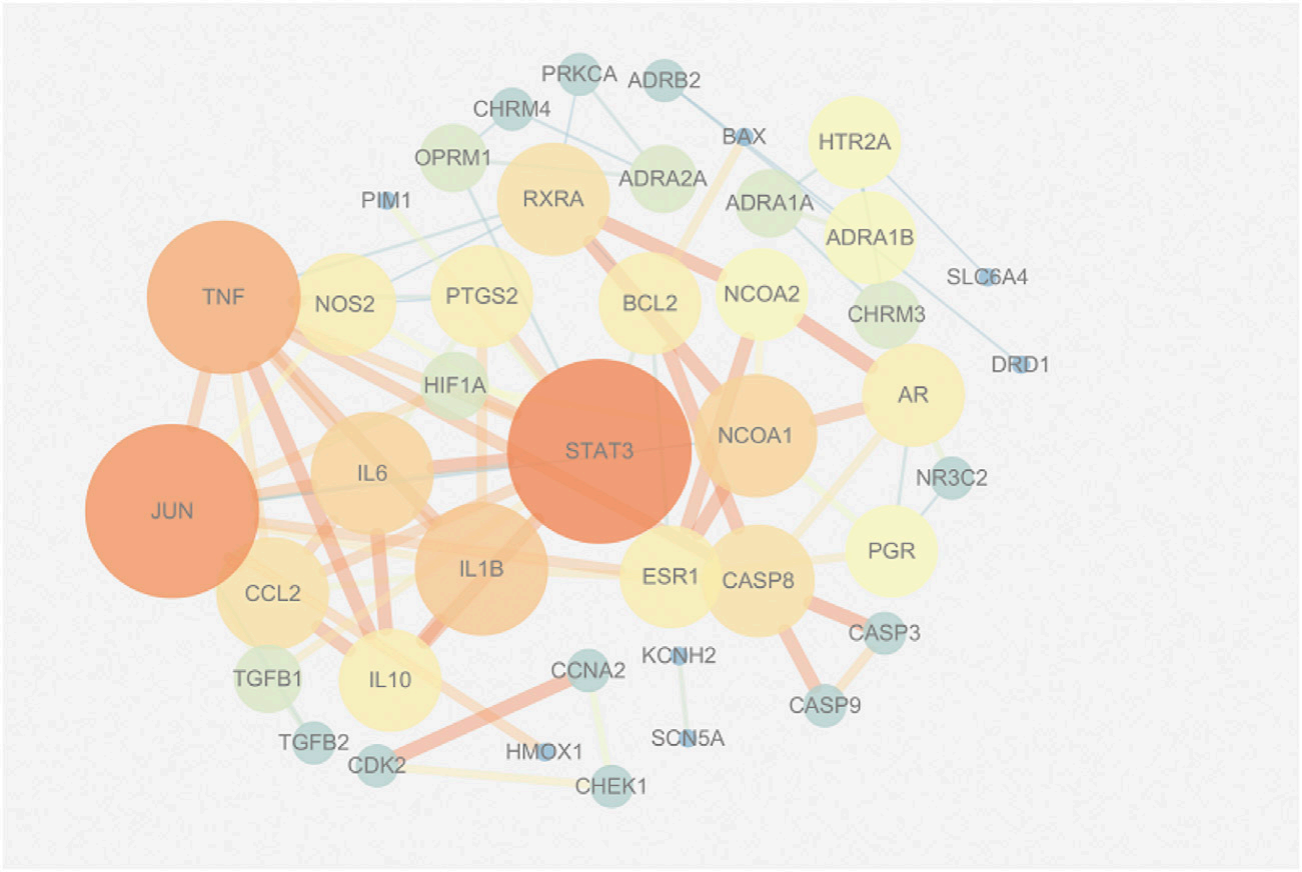
The protein-protein interaction (PPI) network.

This study devised a PPI network of overlapping targets. As illustrated in Figure 2, the network is comprised of 42 nodes and 166 edges. Accordingly, proteins are connectable with other proteins. Moreover, the proteins exhibit a higher number of interactions between themselves than predicted for an arbitrary group of proteins of identical size drawn from the genome. The enrichment is an indicator that at the very least, the proteins are partly linked biologically as a group, and the four proteins STAT3, TNF, JUN, and IL-1β are located at the core position.

### *A. dahurica* Improved Wound Healing in diabetic Mice

**FIGURE 3:**
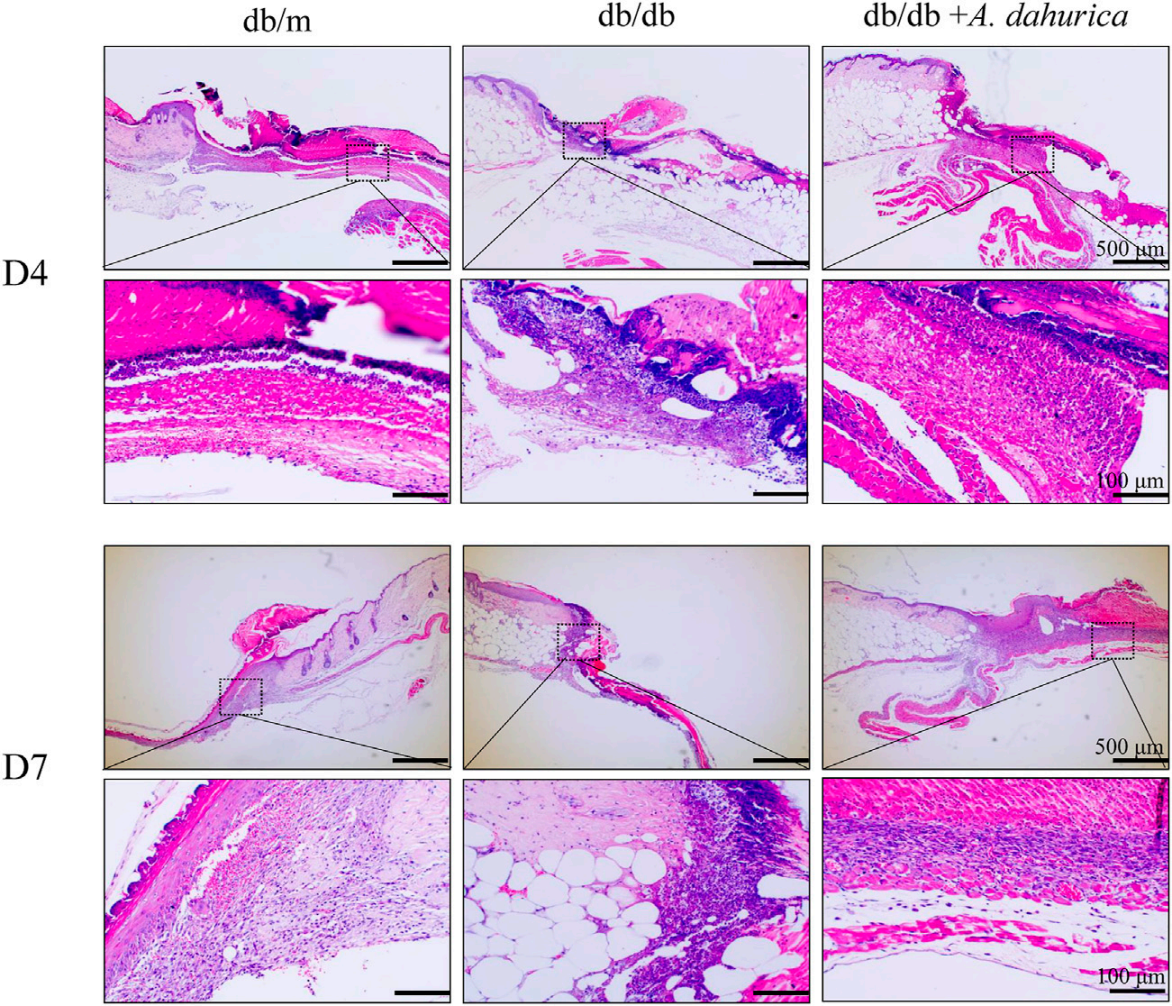
Representative images of H and E staining sections of wounds at days 4 and days 7 post-operation (scale bar: 500, 100 μm), *n* = 6 animals per group.

As shown in Supplementary Figure S2A **,** on day 14 after injuring the mice, the wound regions treated with *A. dahurica* were remarkably narrower than those in db/db mice. Additionally, as shown in Supplementary Figure S2B, *A. dahurica* treatment advanced wound closure in db/db mice at a faster rate than that of db/db mice. As illustrated in Figure 3, in contrast with day 7, the H&E staining related to each wound bed on day 4 exhibited noticeable infiltration of the inflammation. In contrast with the db/db group, there was lesser inflammatory cell infiltration and higher neovascularization in the *A. dahurica* group on day 4. Moreover, H&E staining related to the sections of the wounds exhibited a higher thickness of the granulation tissue in the *A. dahurica*-treated group on day 7 when contrasted with the db/db group. Supplementary Table S1 describes the physical and biochemical parameters of experimental mice after the intervention.

### *A. dahurica* regulates macrophage polarization, reduces inflammation and promotes angiogenesis *in vivo*.

**FIGURE 4:**
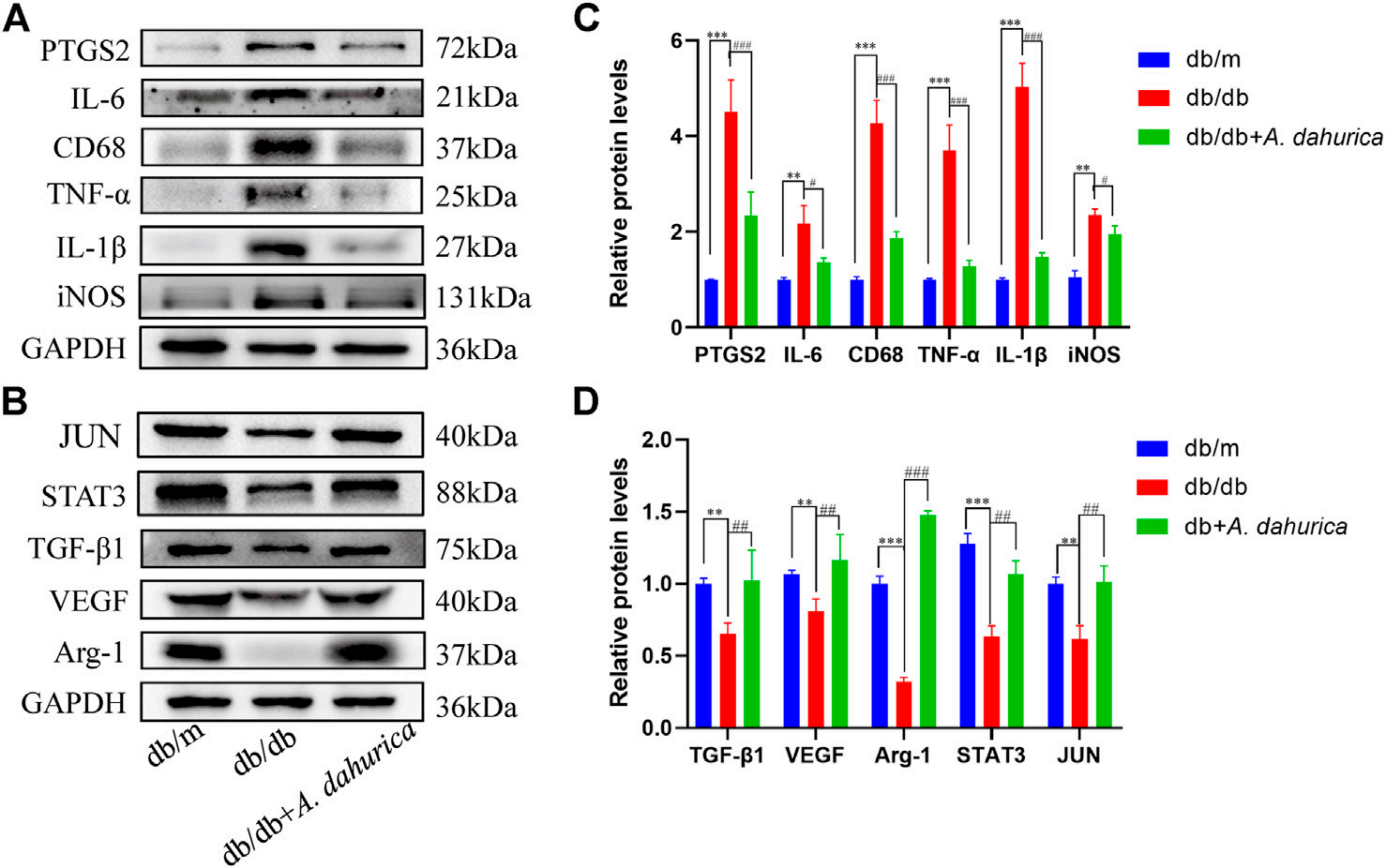
**(A)**: Representative Western blot analysis of PTGS2 (72 kDa), IL-6 (21 kDa), CD68 (37 kDa), TNF-α (25 kDa), IL-1β (27 kDa), and iNOS (131 kDa) in mice. **(B)**: Representative Western blot analysis of Arg-1 (37 kDa), VEGF (40 kDa), TGF-β1 (75 kDa), STAT3 (88 kDa), and JUN (40 kDa) in mice. **(C)**: Densitometric results of PTGS2, IL-6, CD68, TNF-α, IL-1β and iNOS in mice as determined by Western blot. Error bars represent SD. **p < 0.01, ***p < 0.001, ^#^ *p* < 0.05, ^###^ *p* < 0.001, *db/db vs. db/m group, ^#^ db/db *+ A. dahurica* vs. db/db group, n = 6 animals per group. **(D)**: Densitometric results of Arg-1, VEGF, TGF-β1, STAT3, and JUN in mice as determined by Western blot. Error bars represent SD. **p < 0.01, ***p < 0.001, ^##^ *p* < 0.01, ^###^ *p* < 0.001, * db/db vs. db/m group, ^#^ db/db *+ A. dahurica* vs. db/db group, *n* = 6 animals per group.

**FIGURE 5:**
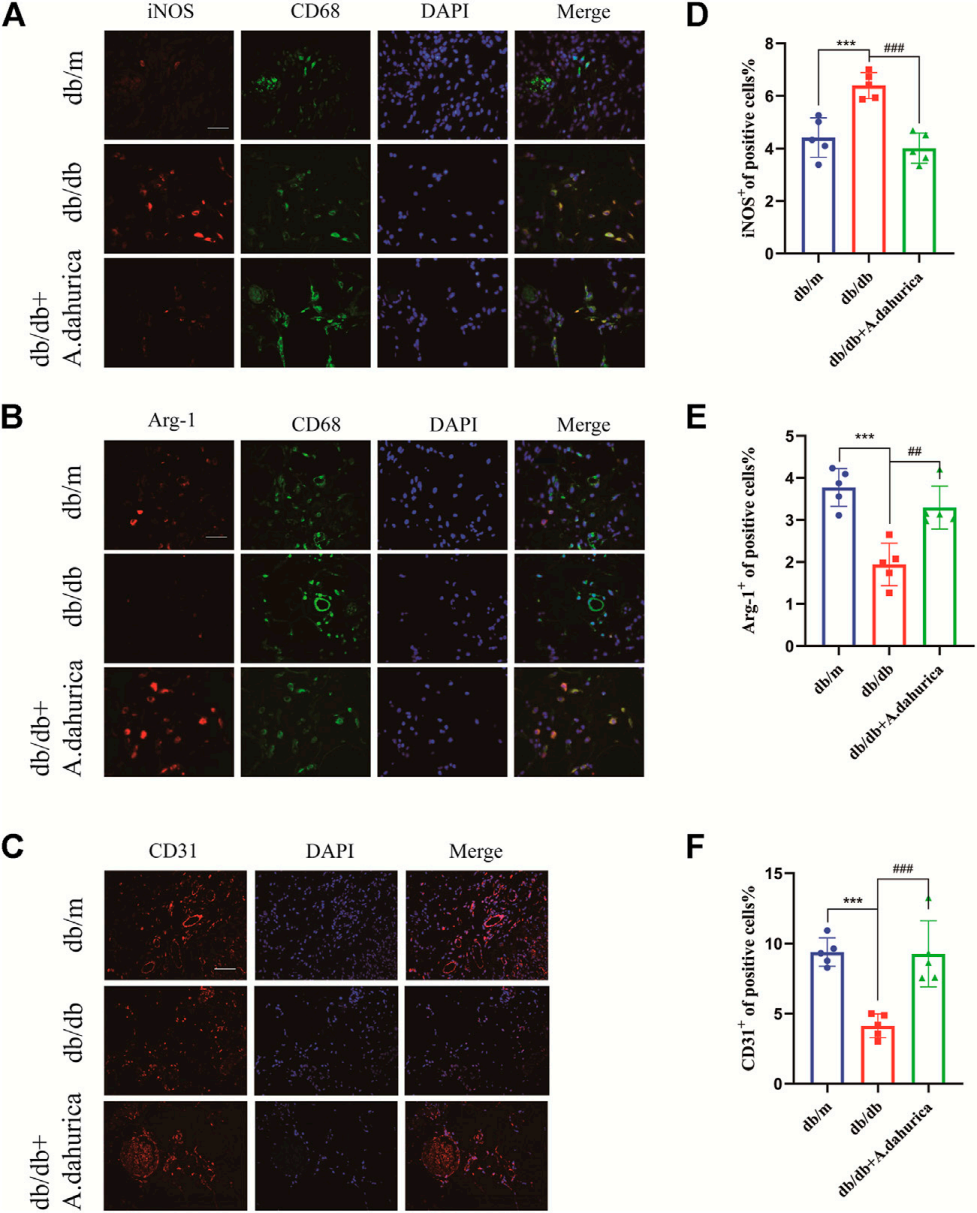
**(A)**: Immunofluorescence staining of iNOS (red) and CD68 (green) in the wound bed (scale bar: 50 μm). **(B)**: Immunofluorescence staining of Arg-1 (red) and CD68 (green) in the wound bed (scale bar: 50 μm). **(C)**: Immunofluorescence staining for CD31 in the wound vessels (scale bar: 50 μm). **(D–F)**: Quantification of iNOS + cells, Arg-1+cells, and CD31 + cells in diabetic skin wounds. (*n* = 5 in each group). ***p < 0.001, ^##^ *p* < 0.01, ^###^ *p* < 0.001, * db/db vs. db/m group, ^#^ db/db *+ A. dahurica* vs. db/db group.

Furthermore, this study also inspected the protein levels of iNOS and Arg-1, they are respectively important markers of M1 and M2 macrophages. According to the Western blot analysis, compared with db/db group in skin tissue, the protein levels of Arg-1 exhibited a significant increment and iNOS exhibited a significant reduction in the *A. dahurica* group. Moreover, compared with the experimental control groups, the protein levels of CD68, IL-1β, TNF-α, and IL-6 were significantly lower in the *A. dahurica* group. In contrast with the db/db mice, the protein level of PTGS2 was significantly decreased in the *A. dahurica* group. The expression level of VEGF and TGF-β1 protein in the skin tissue in the group of *A. dahurica* was significantly increased than that of the db/db mice. Additionally, compared with db/db mice, the levels of protein of STAT3 and JUN were significantly higher in the *A. dahurica* group. (Figures 4A–D). Based on the outcomes of immunofluorescent staining in wounds of *A. dahurica* group, M1-like (iNOS + CD68^+^) cells were significantly reduced and M2- like (Arg-1+ CD68^+^) cells were considerably increased when compared with db/db group wounds on day 4 following the wounding (Figures 5A,B,D,E). Also, this study performed immunostaining on the endothelial cell marker CD31 (Figures 5C,F) to observe the wound neovascularization and the density of CD31^+^ expression on the surface of the wound in the *A. dahurica* group was tremendously elevated compared to untreated db/db mice.

## Discussion

This study indicated a novel mechanism of the *A. dahurica* that accelerates diabetic wound healing through a network pharmacology and experimental validation. According to the outcomes of the study, there were 19 active components that acted on key targets, such as PTGS2, IL-1β, TNF, STAT3, respectively. Moreover, it was speculated that *β*-sitosterol would be related to PTGS2, IL-1β, IL-6, TNF, SATA3, which indicates than an exclusive active ingredient has the capacity to act on several markers for diseases. A previous study found that *β*-sitosterol stimulated the motility of the endothelial cells in the umbilical vein of humans in an *in vitro* wound migration assay (Moon et al., 1999). Besides, stigmasterol has shown effective outcomes when used to heal wounds. The therapeutic effect of stigmasterol on wound healing could be realized by inhibiting the fibroplasia and inflammatory cells (Pinto et al., 2016). However, in the present study, these two components were not identified in the extract of *A. dahurica* by UPLC/Q-TOF-MS system. Imperatorin (BZ3), one of the main active ingredients of *A. dahurica* (Mi et al., 2017) could play an instrumental role in the network. It was speculated that imperatorin would be related to PTGS2, IL-1β, IL-10, IL-6, etc. A past research verified the anti-inflammatory effects of imperatorin on psoriasiform dermatitis in mice (Tsai et al., 2020), which is consistent with our network pharmacology predication.

Meanwhile, the same target was linked to a range of active ingredients in *A. dahurica*, implying that the active ingredients in *A. dahurica* induce a synergistic effect when used to treat wound healing. PTGS2, namely COX-2, is one of the predicted targets, it could play a vital role in wound healing. Reportedly, PTGS2 is a rate-limiting enzyme regulating the synthesis of prostaglandins during the metabolism of arachidonic acid (Kaur et al., 2019), and it is primarily entailed in a range of inflammatory reactions in the body (Sharma et al., 2019). A previous study has also suggested that the PTGS2 inhibitor normalizes the preliminary inflammatory response and proceeds to accelerate the fate of wound healing postinjury (Darby and Weller, 2017). According to recent studies, selective PTGS2 inhibitors enhance the quality of post-wounding scars (Hu et al., 2014), which aligns with the outcomes predicted in the network.

Furthermore, the PPI network exhibited sophisticated interactions between the 54 overlapping genes. STAT3, JUN, TNF and IL-1β are located at the core position and had close interactions, this enrichment implies that in the very least, the proteins are partly connected biologically as a group, which regulates the advancement of diabetic wound healing (Wu et al., 2020). Reportedly, STAT3 has central roles in healing skin wounds, keratinocyte migration, and growth of hair follicles (Sano et al., 2008). The present *in vivo* study discovered a significant increase in the protein level of STAT3 after *A. dahurica* intervention. The result indicates that STAT3 may be the target of *A. dahurica*, which is consistent with the prediction of the PPI network. In addition, in the present study, western blot data revealed that *A. dahurica* significantly increased JUN expression on the protein levels. It has been demonstrated that c-JUN is crucial for regulating an epidermal growth factor autocrine loop, which creates the epidermal leading edge. Thereby, it has a critical role in the cutaneous healing of wounds (Li et al., 2003). Another research reported by Yue et al. found that the overexpression of c-JUN improves the rapidity at which wounds heal in diabetic rats (Yue et al., 2020). Accordingly, the results suggest that *A. dahurica* may improve the rate at which wounds heal through several targets, is consistent with the idea of TCM. Moreover, TNF and IL-1β also have a significant part to play in the network, inferring that *A. dahurica* may promote diabetic wound healing by regulating the inflammatory response.

Macrophage polarization assumes critical regulatory roles during inflammatory and in the initial proliferative phase of wound healing (Witherel et al., 2021). The chief human macrophages were polarized to distinct phenotypes, which include pro-inflammatory M1, pro-healing M2, and a hybrid M1/M2, all are produced through the simultaneous exposure of macrophages to M1-and M2-promoting parallel stimuli. Generally, the inflammatory phase of normal wounds takes anywhere from several hours to 3–4 days (Hesketh et al., 2017). However, the diabetic wound environment is characterized by pro-inflammatory M1 phenotype hyperpolarization and extended inflammation due to the inhibited advancement in the healing process and then cause the development of diabetic foot ulcers in humans (Ganesh and Ramkumar, 2020). A previous study reported that *A. dahurica* inhibited the expression of PTGS2 and iNOS in lipopolysaccharide (LPS)-stimulated RAW264.7 cells (Lee et al., 2011). Having said so, the exact underlying mechanism of *A. dahurica* that acts on macrophages in diabetic wound healing is yet to be fully established. Therefore, this study conducted *in vivo* experiments to ensure the validity of the prediction to a certain degree by verifying the role that *A. dahurica* plays in regulating the polarization of macrophages. The present study found that *A. dahurica* therapy advanced excisional wound healing in a full-thickness cutaneous wound healing model in db/db mice. Also, *A. dahurica* inhibited M1 polarization of ulcer tissue macrophages in db/db mice and promoted M2 polarization. M1 macrophages are a critical source for a range of inflammatory factors in wounds. Following *A. dahurica* treatment, the secretion of inflammatory factors, such as IL-1β, TNF-α, and IL-6 also reduced significantly, which is of great significance to diminish wound inflammatory cell infiltration. This finding is consistent with previous studies that indicated *A. dahurica* decreased inflammatory cytokines levels, such as TNF-α, IL-6, and accelerated wound closure in rats (Yang et al., 2020). Furthermore, a recent study reported that *A. dahurica* decreased the mRNA expression of IL-6, IL-4, and TNF-α in the tissues of mice skin. It generated beneficial effects of atopic dermatitis (Ku et al., 2017), which conforms with the findings of the present study related to the anti-inflammatory effects of *A. dahurica*. Moreover, there was a significant reduction in the level of protein of PTGS2 following *A. dahurica* intervention, which was consistent with the network pharmacological prediction. Similarly, numerous studies have reported the anti-inflammatory effects of imperatorin (BZ3), a main bioactive component of *A. dahurica*, such as reduced iNOS and PTGS2 expression, as well as IL-6 and TNF-α production (Huang et al., 2012; Li et al., 2019; Wang et al., 2021), which is consistent with our network predication. Guo et al. also discovered that imperatorin significantly reduced the TNF-α levels induced by LPS, IL-1β, and IL-6 in RAW 264.7 macrophages in a concentration-dependent approach (Guo et al., 2012). In addition, as reported by Moon et al. (Moon et al., 2008), isoimperatorin (BZ4), another active ingredient of *A. dahurica*, inhibits the PTGS2 generation in bone marrow-derived mast cells (BMMC). Therefore, it could be a novel anti-inflammatory drug, which is consistent with the results projected in the network. Besides, our network pharmacology results are also consistent with a study that showed the anti-allergic inflammation effects of oxypeucedanin hydrate (BZ10) in RBL-2H3 cells (Li and Wu, 2017).

Moreover, *A. dahurica* treatment also exhibited a clear enhancement of angiogenesis to accelerate wound closure. VEGF refers to a highly specific growth factor that promotes vascular endothelial cell growth secreted by M2 subtype macrophages (Ning et al., 2019). The present study also found that after *A. dahurica* gavage, M2 subtype macrophages of db/db mice secreted a significant amount of VEGF in the later stage of inflammation, as a result, it could advance the proliferation, migration, and angiogenesis of endothelial cells in granulation tissue. Likewise, a recent study by our group demonstrated that compared to untreated mice, *A. dahurica*-treated diabetic mice had a higher number of freshly formed vessels during the proliferative phase (Zhang et al., 2017). Additionally, the present study also indicated that compared to the db/db mice, the TGF-β1 expression was remarkably higher in the group treated with *A. dahurica*. It suggests that wound healing necessitates TGF-β1, a cytokine that effectively triggers fibroblasts (Gan et al., 2019). Besides, in patients with diabetes, the resting skin is thinner in the panniculus adiposus layer and hair follicles are reduced (McBride et al., 2014). However, our research primarily concentrates on the inflammatory response related to diabetic wound healing, which primarily occurs during the initial phase of wound healing. In future research, we will extensively examine the effect of *A. dahurica* on the skin and hair related to wound healing in diabetes. The results of this study collectively demonstrated that *A. dahurica* shows potential therapeutic benefits on wound healing. Besides, the inhibition of inflammation is most likely to be one of the preferred approaches to accelerate wound healing, particularly by regulating the polarization of macrophages, which aligns with the targets projected in the network.

## Conclusion

In summary, this study provided beneficial fresh insights to advance our comprehension of the mechanism underlying the action of *A. dahurica* accelerating diabetic wound healing. Using network pharmacology and *in vivo* experiments, this research systematically revealed that *A. dahurica* may have the capacity to effectively improve the inflammatory response in the diabetic wound healing process. Therefore, the preliminary conclusion is that *A. dahurica* is useful in the treatment of diabetic wound healing by regulating the polarization of macrophages, which paves the path for the specific molecular mechanism of active components in *A. dahurica*. Additionally, the network pharmacology framework explained in this study has the potential to provide a novel platform to treat diabetic wound healing with TCM and for the development of novel and safe anti-inflammatory medication for treating wounds in diabetic patients.

## Data Availability

The raw data supporting the conclusion of this article will be made available by the authors, without undue reservation, to any qualified researcher.
