# Supplementary figures and images for: Angelica Dahurica Regulated the Polarization of Macrophages and Accelerated Wound Healing in Diabetes: A Network Pharmacology Study and In Vivo Experimental Validation
Source: Front Pharmacol. 2021 Jun 21;12:678713. doi: 10.3389/fphar.2021.678713 (PMC8256266; doi:10.3389/fphar.2021.678713)

Arg-1


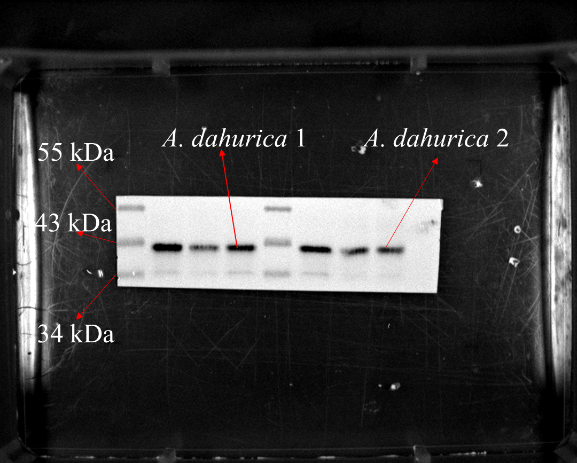




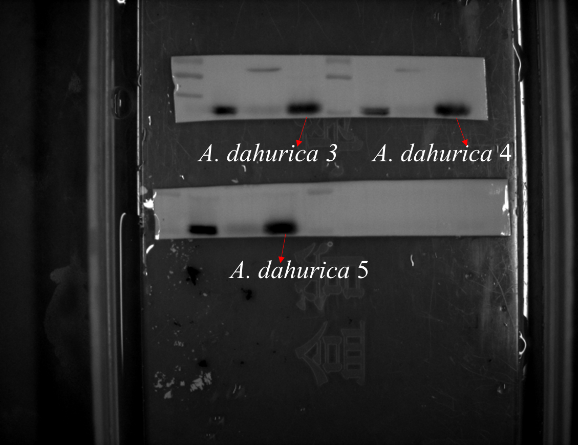




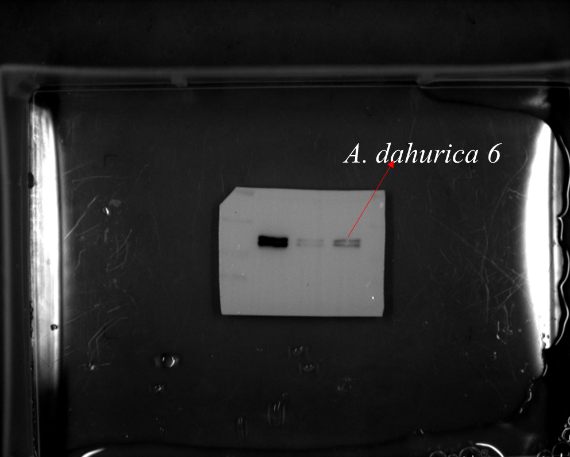




CD68


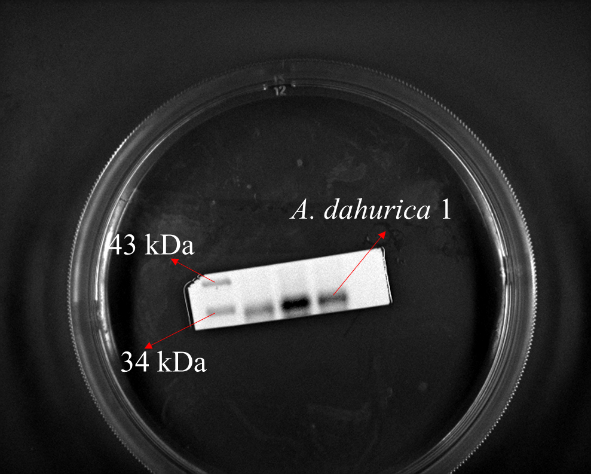




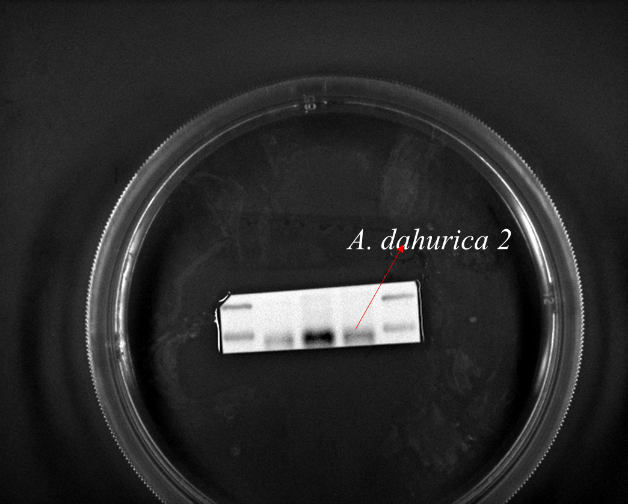




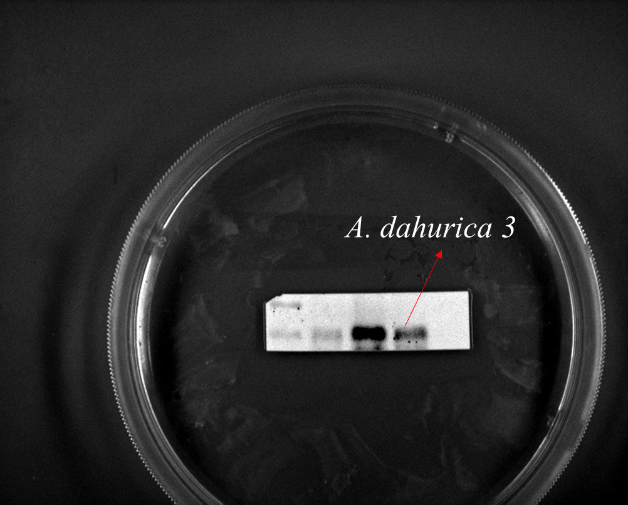




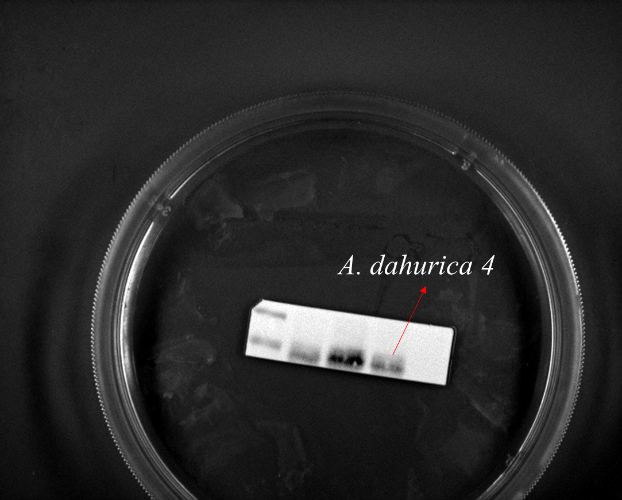




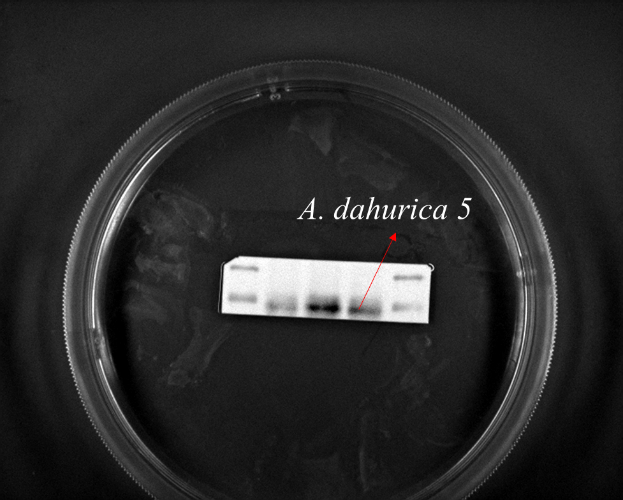




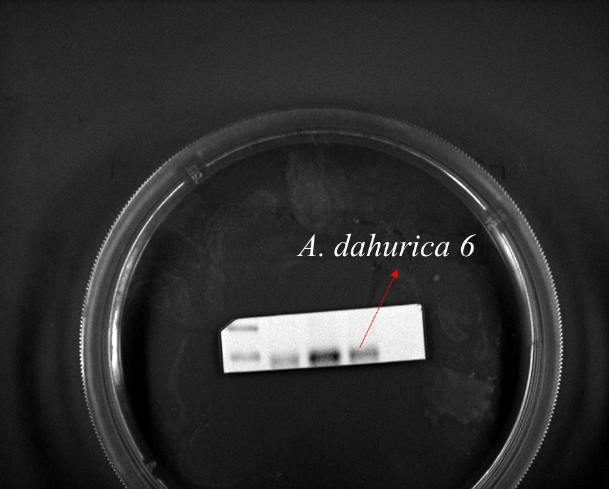




GAPDH


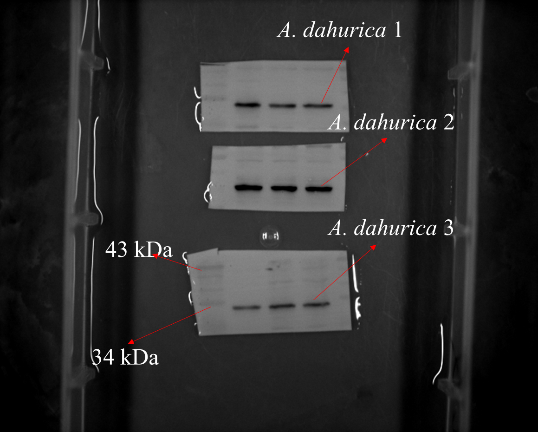




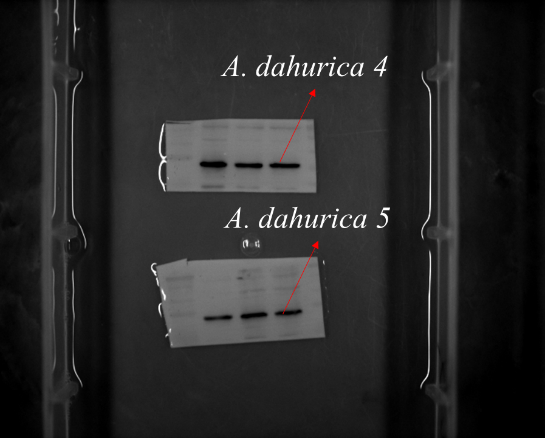




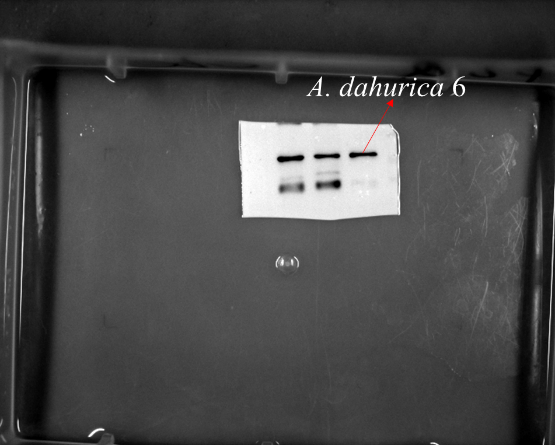




IL-1β


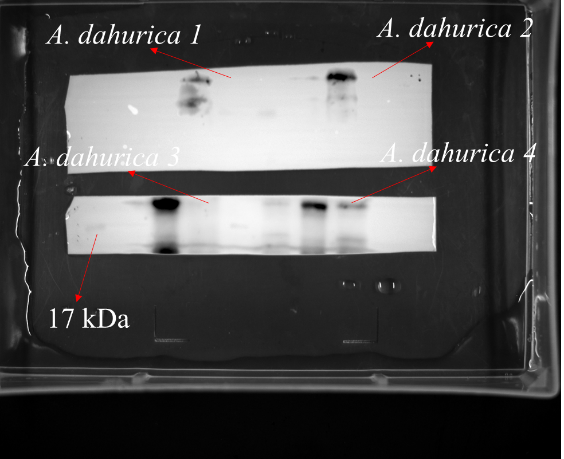

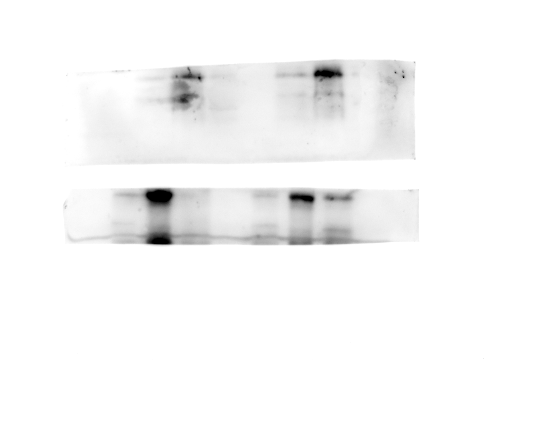


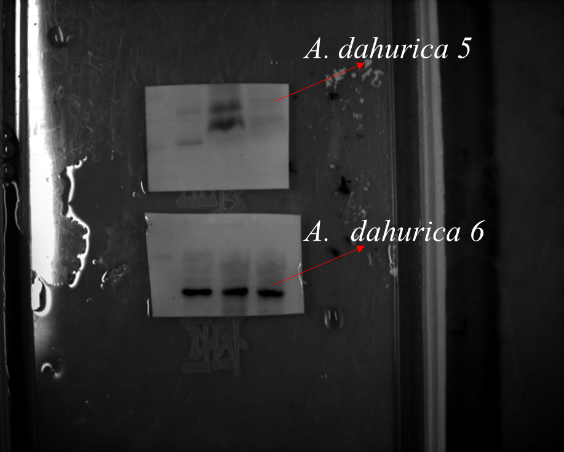




IL-6


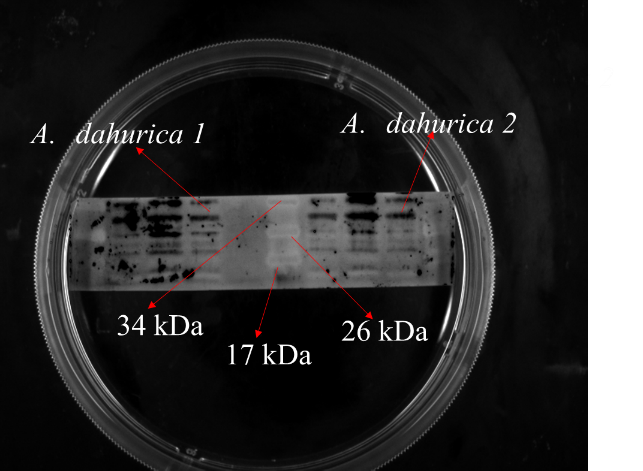




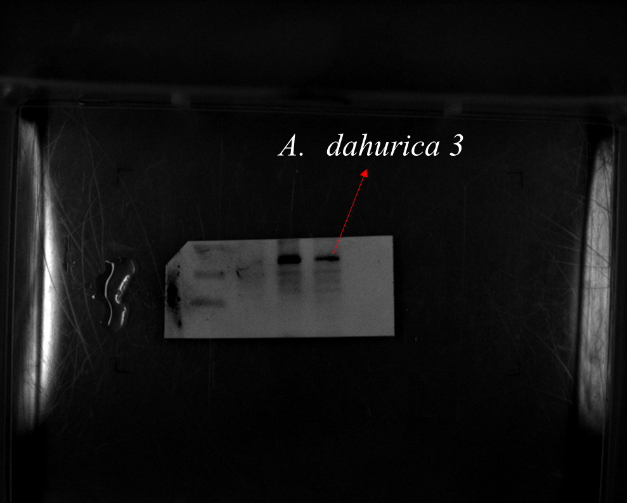




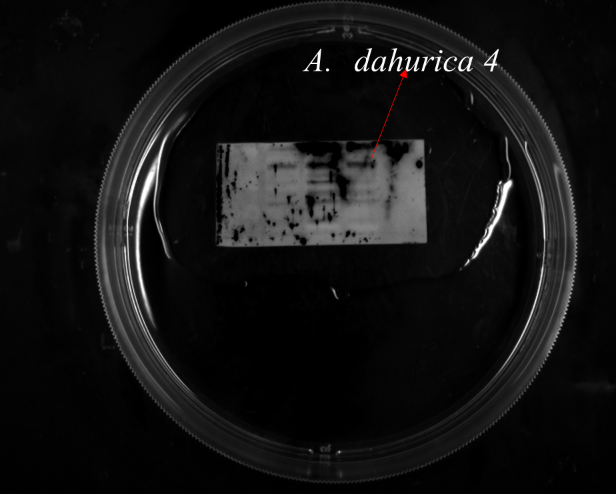




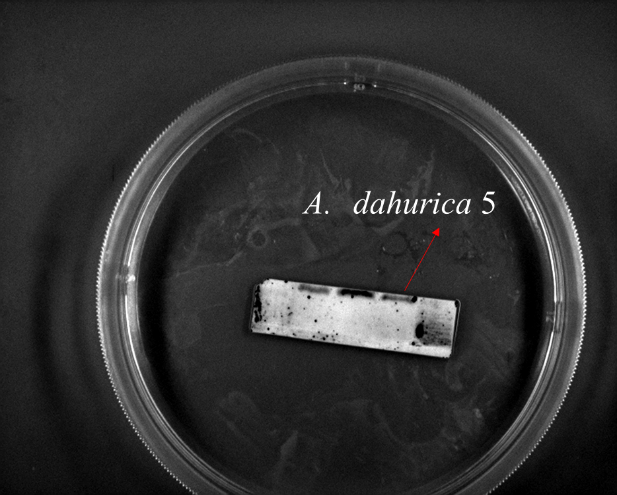




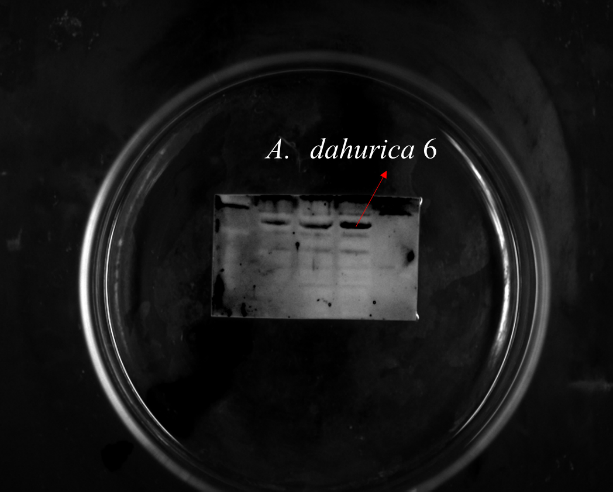




iNOS


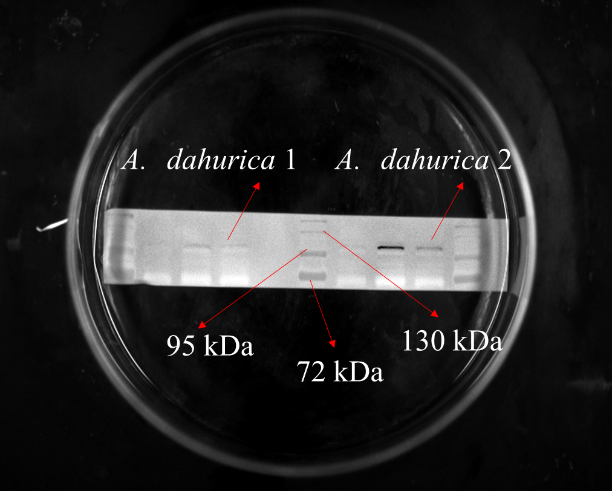




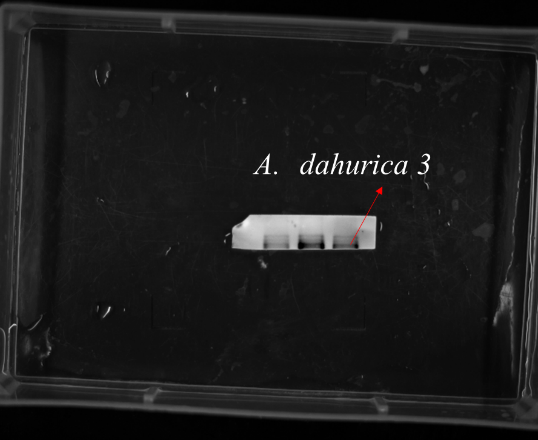

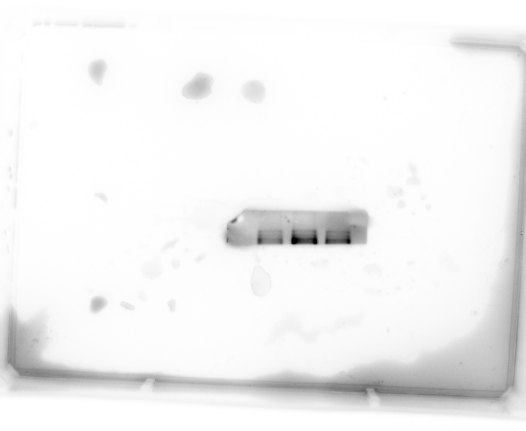


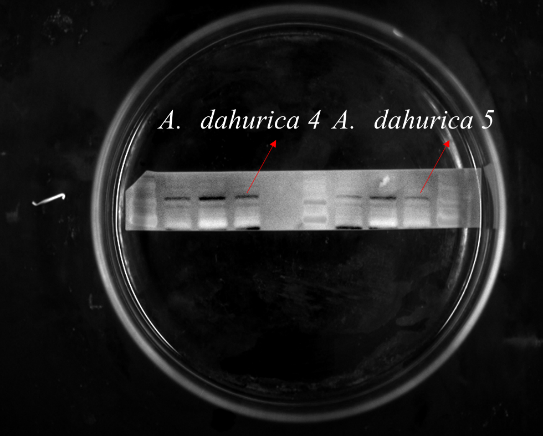




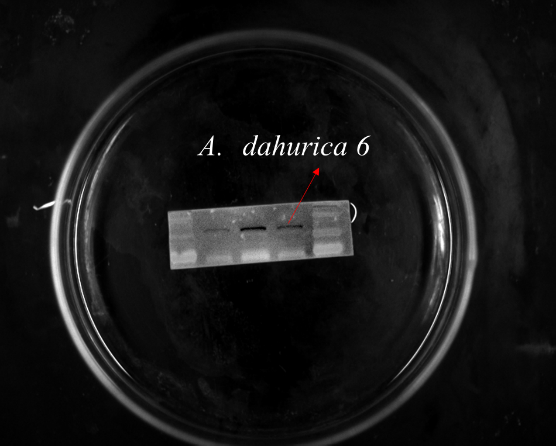




JUN


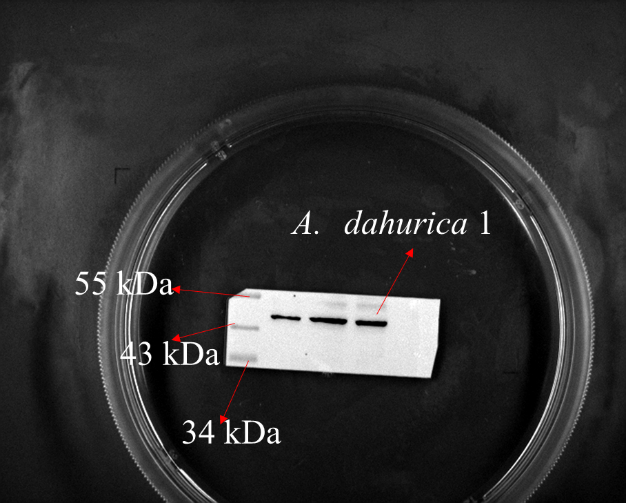




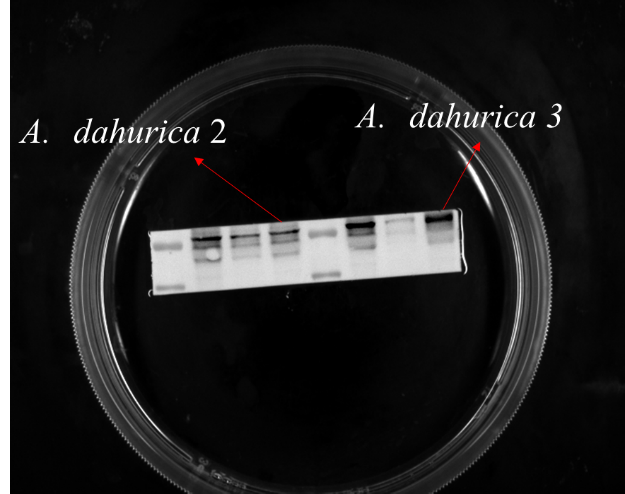




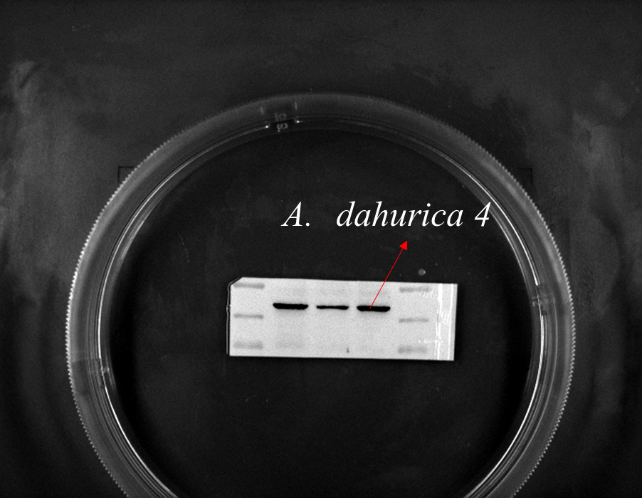




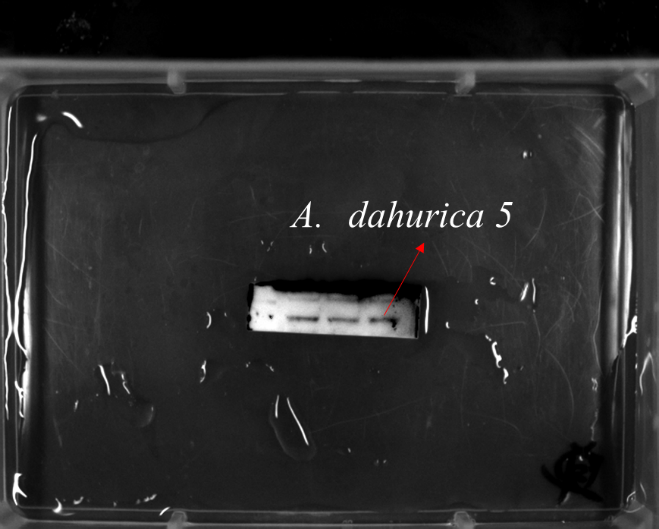




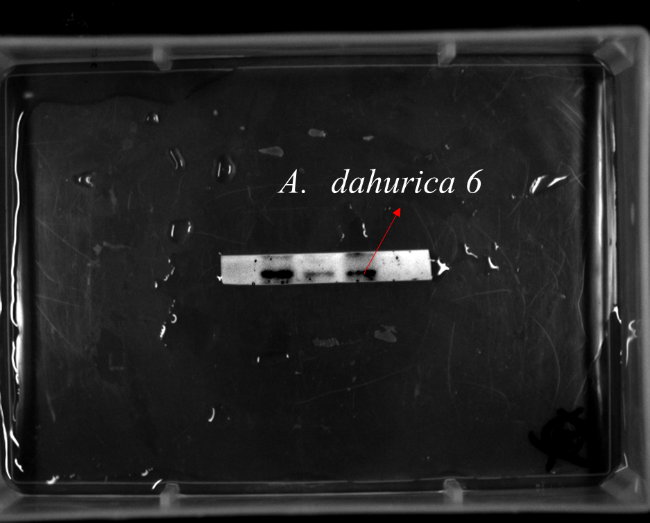




VEGF


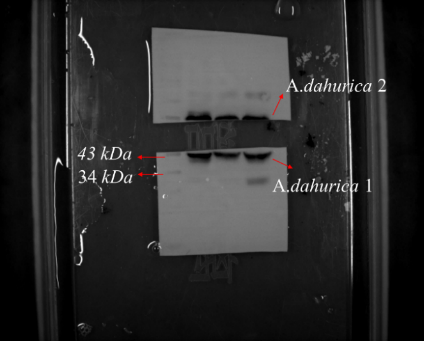




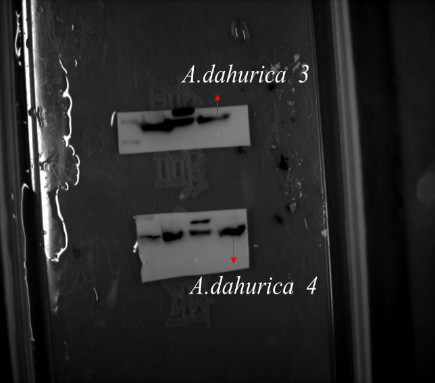




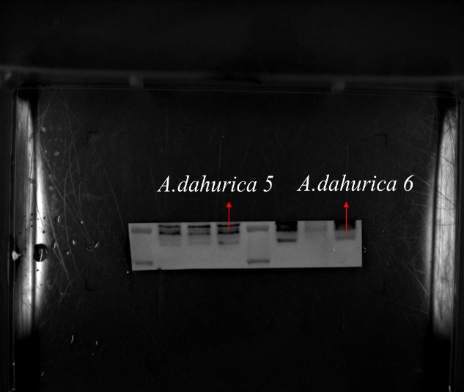




TNF-α


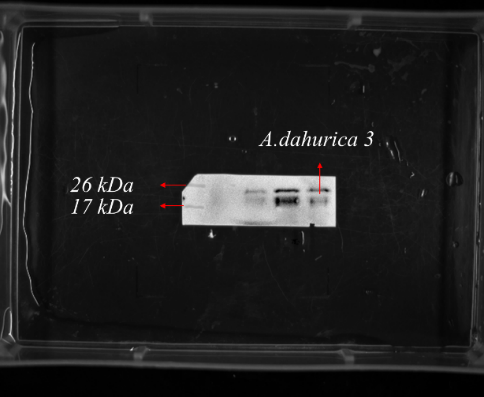

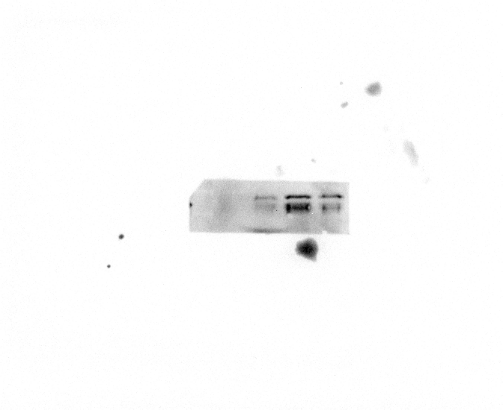


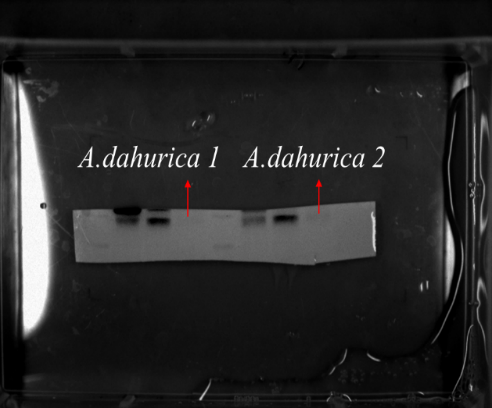

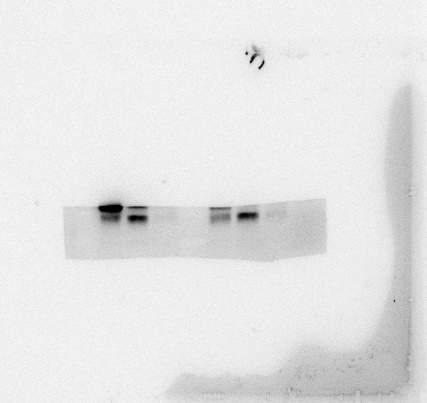


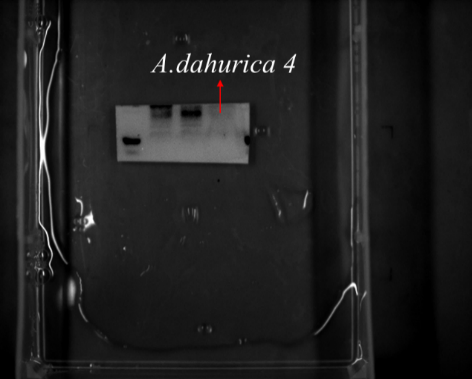




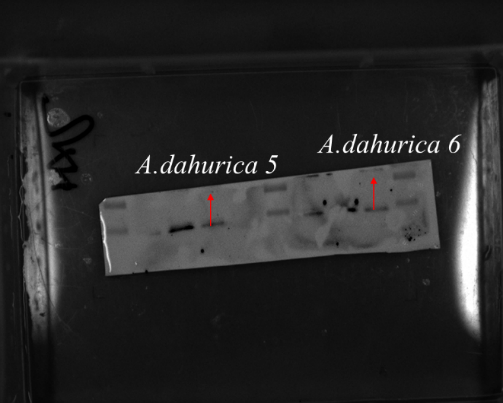

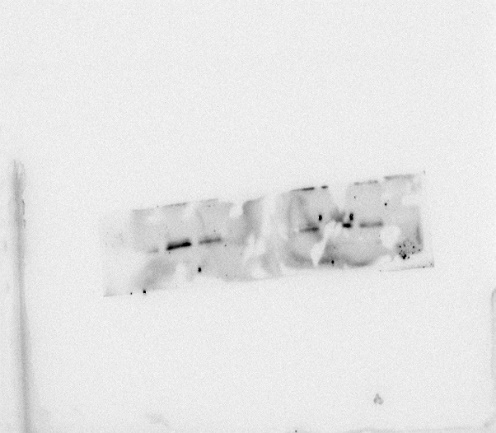


TGF-β1


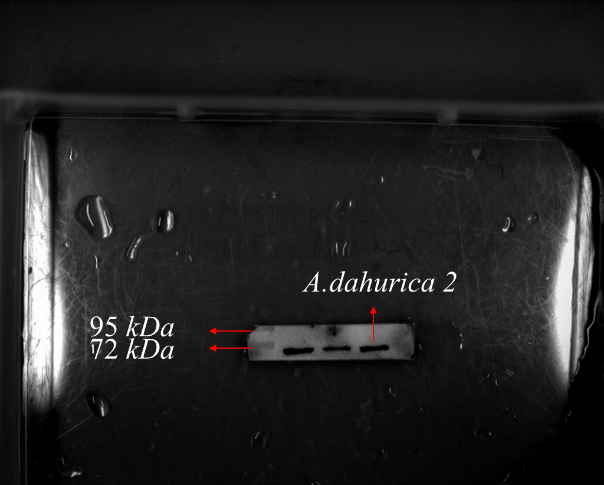

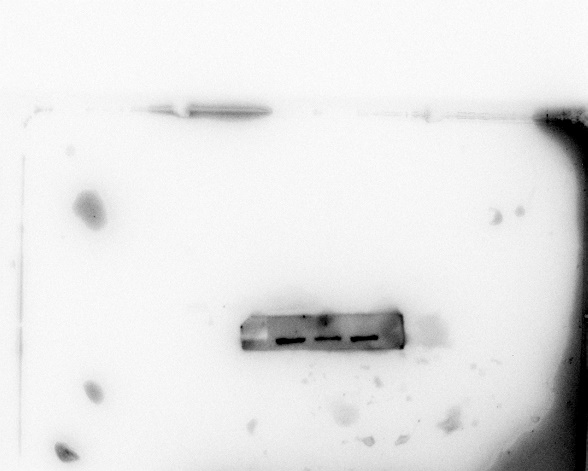


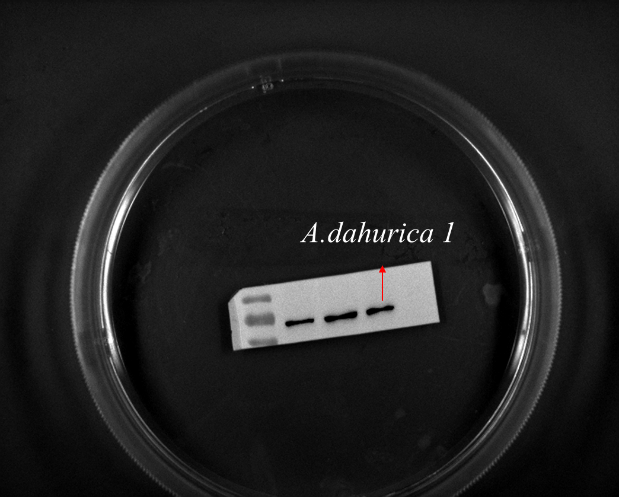




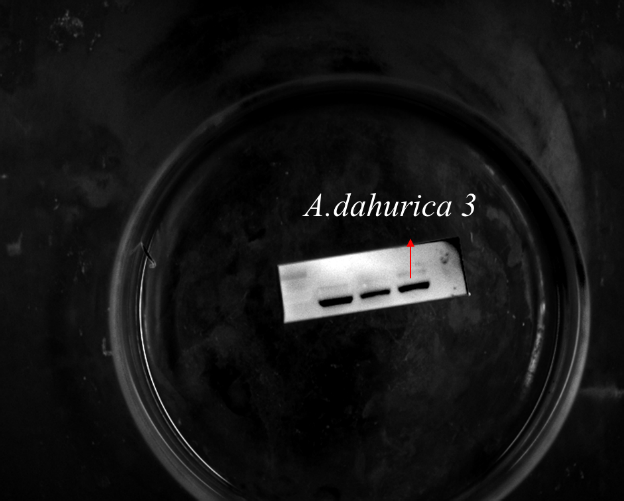

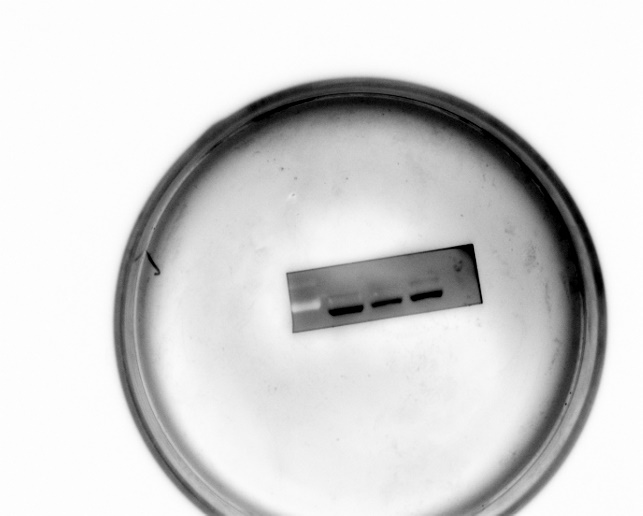


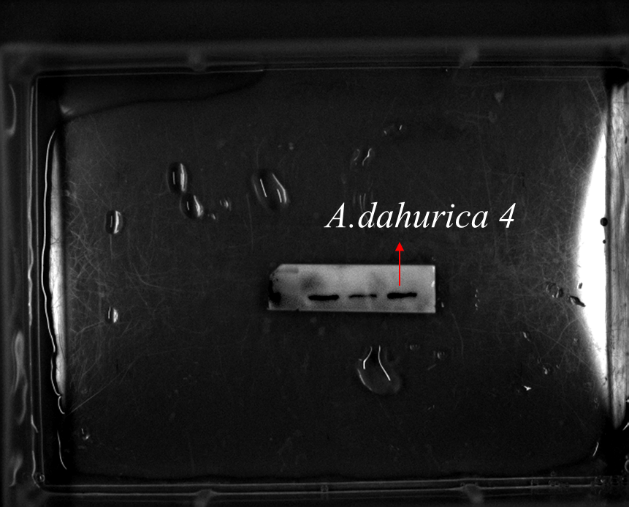




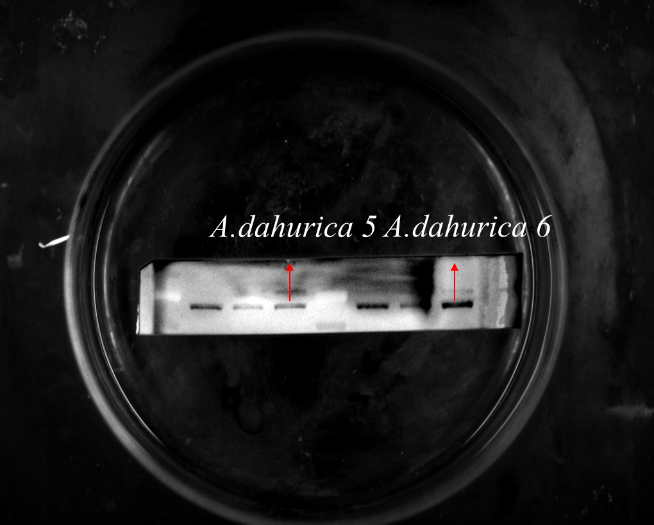

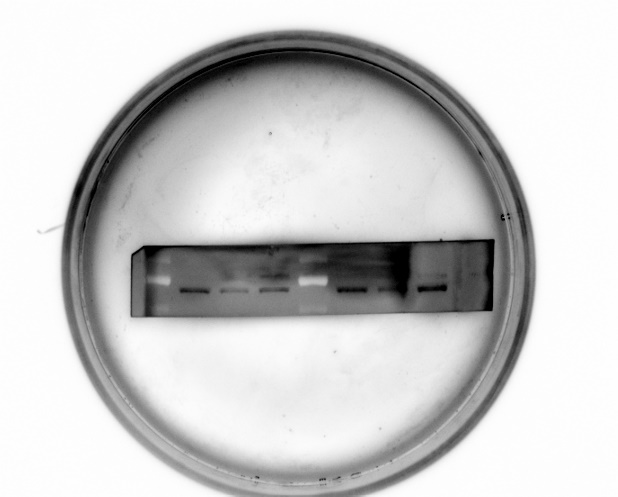


STAT3


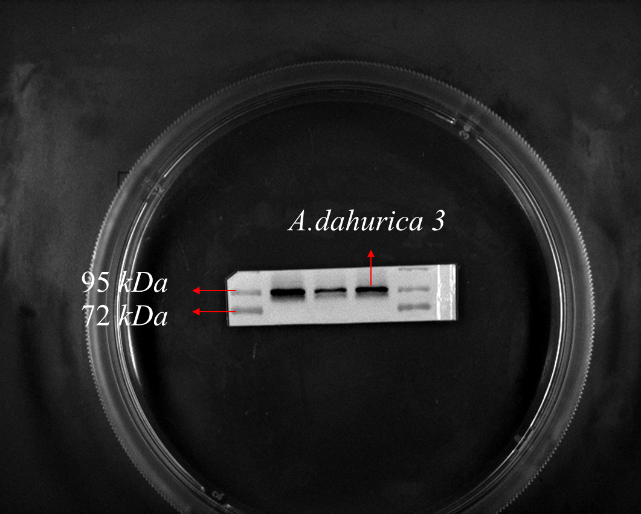




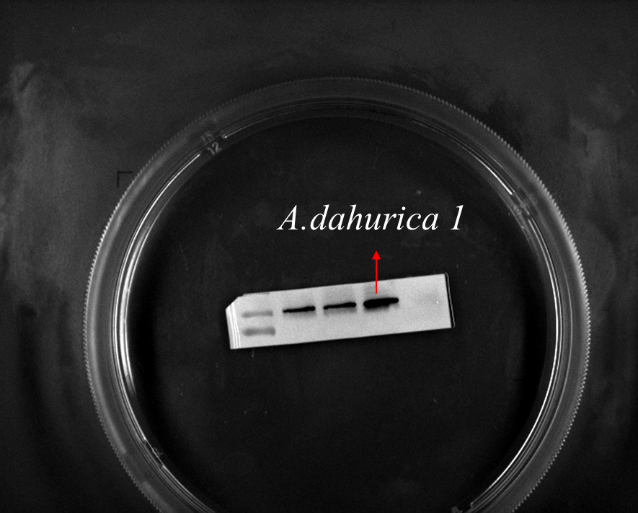




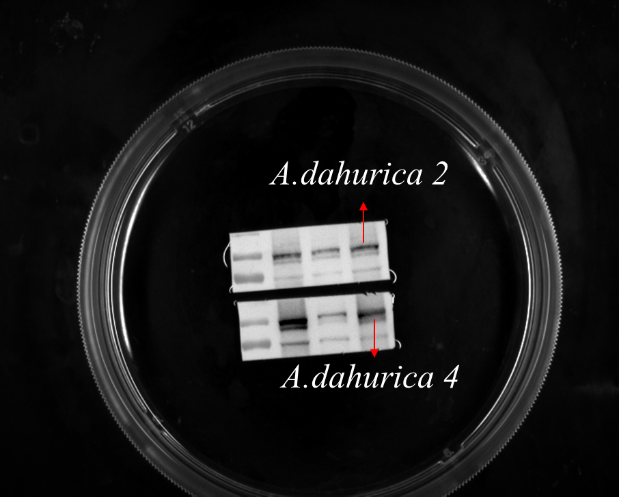




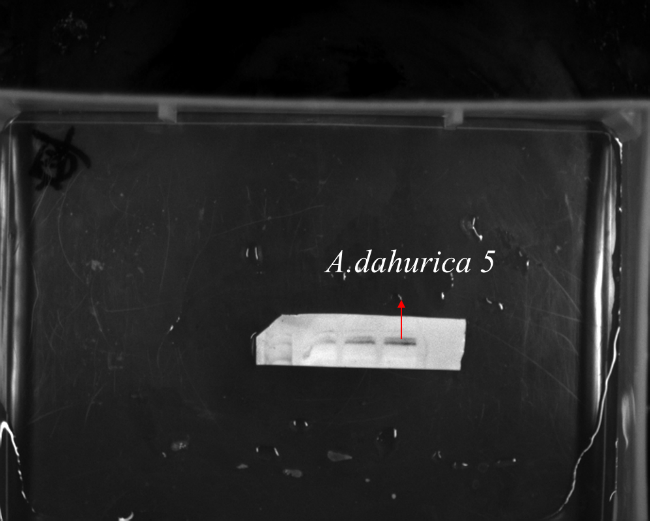




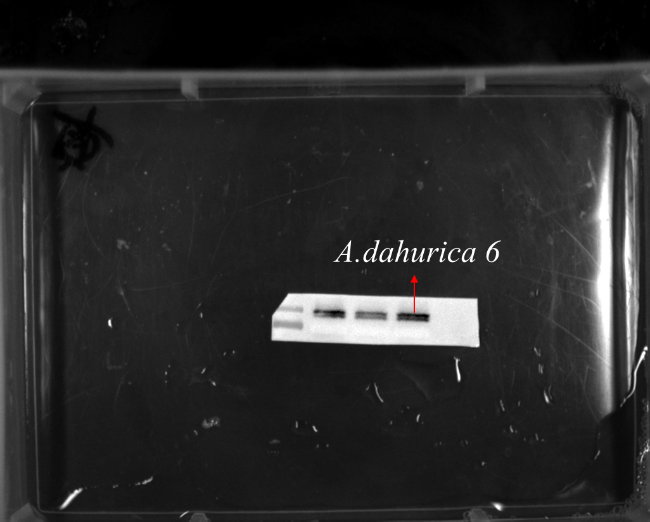




PTGS2


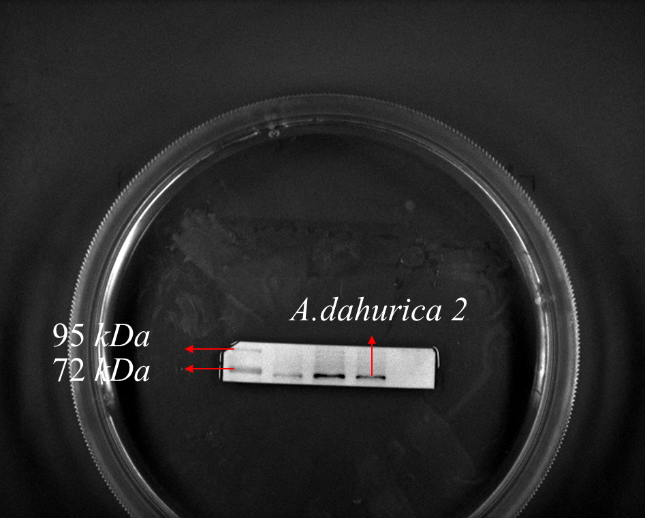




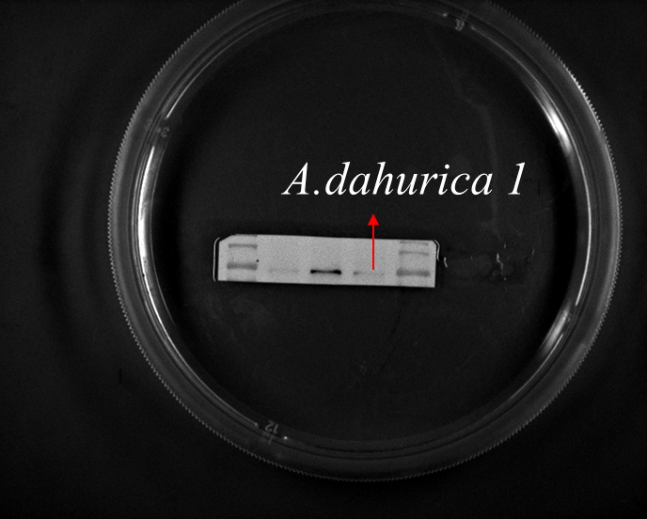



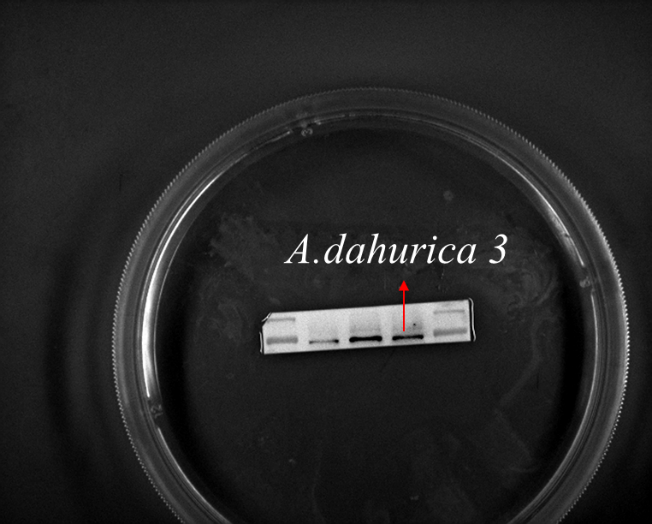




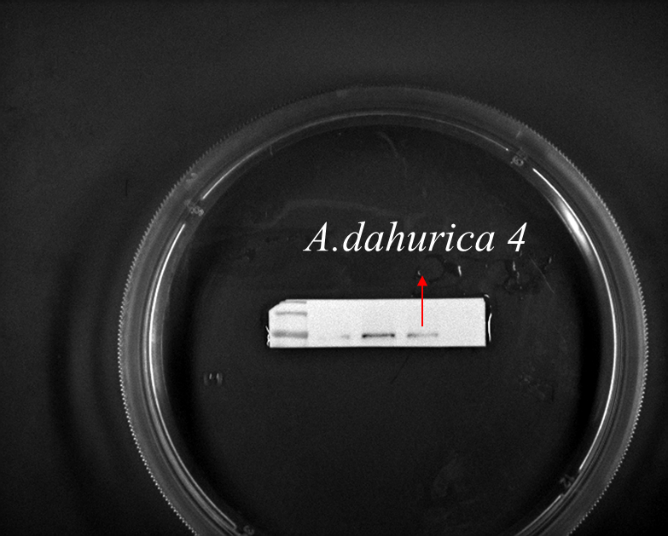




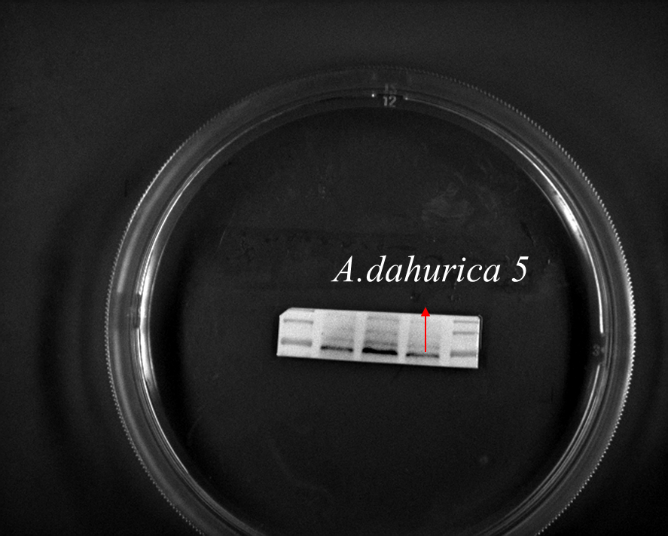

Supplement: Supplementary file 1 [file DataSheet2.docx]
