# Supplementary material for: Angelica Dahurica Regulated the Polarization of Macrophages and Accelerated Wound Healing in Diabetes: A Network Pharmacology Study and In Vivo Experimental Validation
Source: Front Pharmacol. 2021 Jun 21;12:678713. doi: 10.3389/fphar.2021.678713 (PMC8256266; doi:10.3389/fphar.2021.678713)
Supplement: Supplementary file 2 [file DataSheet1.docx]

**Figure S1**


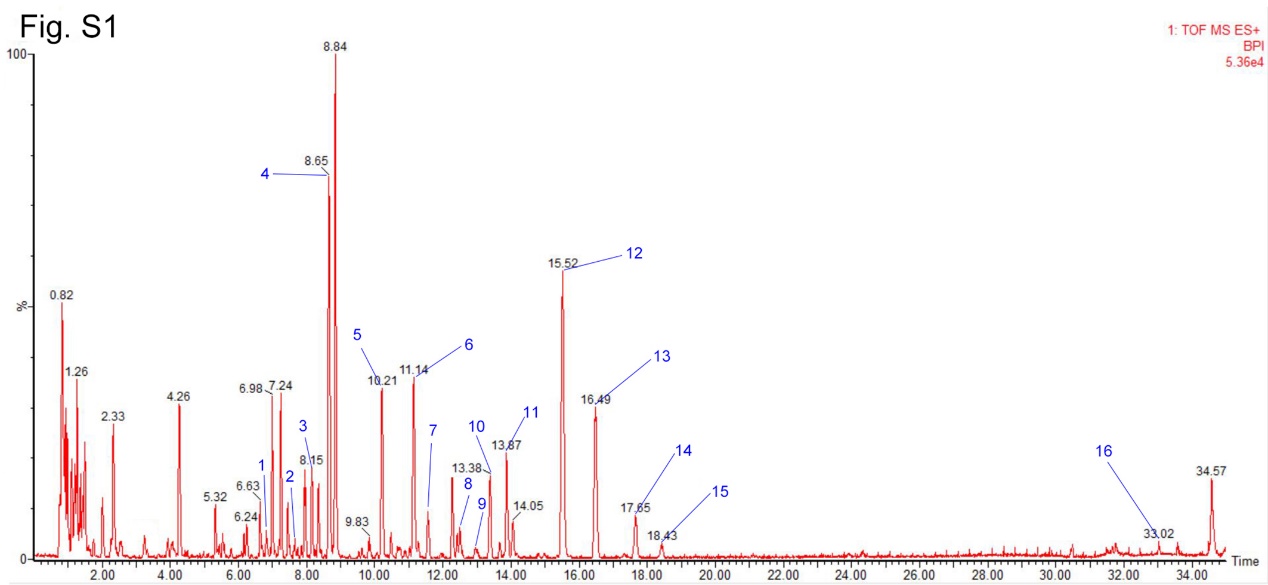


**Figure S1**. The main compounds of the extracts of *A. dahurica*

The main components of the extracts of *A. dahurica* were detected by UPLC/Q-TOF-MS system. According to retention time, molecular weight and ion fragments, 16 of main compounds were identified as Figure S1: 1: Nodakenin, 2: Byakangelicin,

3: Pabulenol, 4: Oxypeucedanin hydrate, 5: Xanthotoxin, 6: Bergapten,

7: oxypeucedanin, 8: 5-methoxy-8-hydroxypsoralen , 9: Neobyakangelicol,

10: Alloisoimperatorin, 11: Isoimperatorin, 12: Imperatorin, 13: Cnidilin, 14: Alloimperatorin, 15: suberosin,

16: Bis[(2R)-2-ethylhexyl] benzene-1,2-dicarboxylate (ZINC03860434).

**Figure S2**


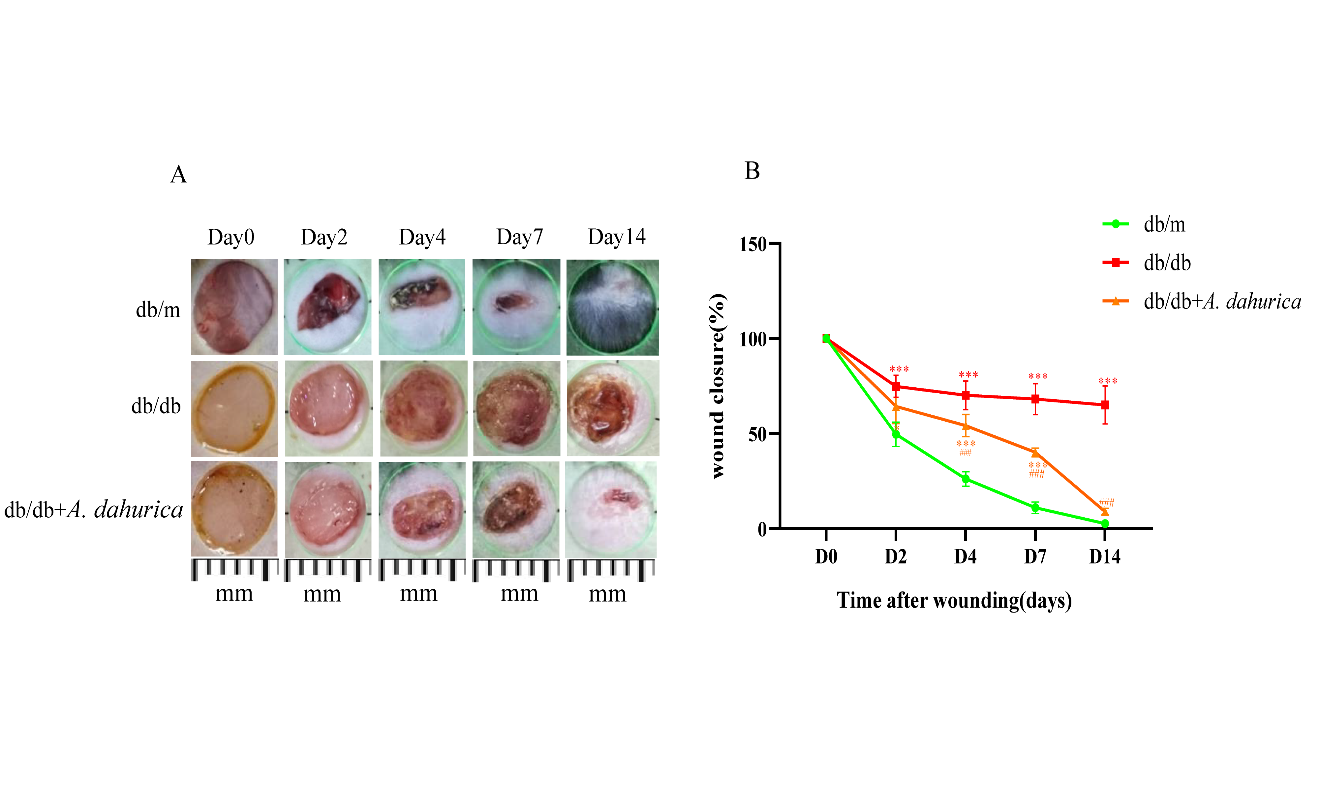


**Figure S2** (A): Representative images of the healing process in *A. dahurica*-treated mice on different days.

(B): The level of wound closure is expressed as a percentage of initial wound area (n = 6). *p < 0.05 versus db/m group, ***p < 0.001 versus db/m group, ^###^p < 0.001 db/db *+ A. dahurica* versus db/db group.

**Table S1**. Physical and biochemical parameters of experimental mice after *A. dahurica* intervention.

|  | db/m | db/db | db/db+*A. dahurica* |
| --- | --- | --- | --- |
| BW (g) | 22.96±0.31 | 45.32±0.43* | 44.27±0.48* |
| FPG (mmol/L) | 6.94±0.24 | 23.06±1.4* | 22.78±1.58* |
| ALT (U/L) | 62.63±3.14 | 65.45±4.04 | 66.35±4.53 |
| AST (U/L) | 170.45±7.65 | 168.48±8.26 | 174.35±7.23 |
| BUN (mmol/L) | 7.45±0.84 | 6.98±0.74 | 7.36±0.63 |
| Scr (umol/L) | 72.23±5.89 | 71.25±4.94 | 70.42±5.14 |

n=6 per group. Data are expressed as the mean ± standard deviation (SD). BW, body weight; FPG, fasting plasma glucose; ALT, alanine aminotransferase; AST, aspartate aminotransferase; BUN, blood urea nitrogen; Scr, serum creatinine; *A. dahurica*, Angelica dahurica. *P < 0.05 vs. the db/m group.
